# MOF-based catalysts for sustainable biodiesel production: classification, performance, and advances from 2020 to 2025

**DOI:** 10.1039/d5ra07154b

**Published:** 2025-12-09

**Authors:** Basem E. Keshta, Sahar Abdalla, Dhiss Tesnim, Yasmeen G. Abou El-Reash, Eida S. Al-Farraj, Mahmoud M. Shaban, Ahmed El-Harairy, H. M. El-Saeed, Amged El-Harairy, Ebtehal A. Shaban, Ahmed S. Abou-Elyazed, Mohamed N. Goda, Elsayed M. Atwa, Amr E. Keshta

**Affiliations:** a Key Laboratory of Ministry of Education for Advanced Catalysis Materials, Zhejiang Normal University Jinhua 321004 P.R. China basem.keshta@science.tanta.edu.eg; b Chemistry Department, Faculty of Science, Tanta University Tanta 31512 Egypt; c Department of Chemistry, College of Science, Imam Mohammad Ibn Saud Islamic University (IMSIU) Riyadh P.O. Box, 90950 11623 Saudi Arabia; d National Engineers School of Gabes, Laboratory of Research: Processes, Energy, Environment & Electrical Systems PEESE (LR18ES34), University of Gabes Tunisia; e Department of Mechanical and Materials Engineering, University of Nebraska-Lincoln Lincoln NE 68588 USA; f Department of Chemical and Biomolecular Engineering, University of Nebraska-Lincoln Lincoln NE 68588 USA ael-harairy2@huskers.unl.edu; g Department of Chemistry, Faculty of Science, Mansoura University Mansoura 35516 Egypt; h Department of Crop and Animal Sciences, Albrecht Daniel Thaer-Institute of Agricultural and Horticultural Sciences, Faculty of Life Sciences, Humboldt-Universität zu Berlin Albrecht-Thaer-Weg 5 14195 Berlin Germany; i Chemistry Department, Faculty of Science, Menoufia University Shebin El-Kom 32512 Egypt; j Agricultural Engineering Research Institute, Agricultural Research Center Giza 12619 Egypt; k Chemistry Department, Faculty of Science, Tanta University Tanta 31512 Egypt; l Smithsonian Environmental Research Center (SERC) Maryland USA

## Abstract

Global energy demand and environmental concerns have intensified the search for renewable fuels, with biodiesel emerging as a sustainable substitute for petroleum diesel. Efficient catalysis remains the bottleneck for large-scale biodiesel production. While heterogeneous catalysts offer advantages of reusability and separation, their performance is limited by stability, active sites availability, and reduced activity under harsh conditions. Metal–organic frameworks (MOFs), with their high surface area, tunable porosity, and structural versatility, have recently attracted increasing attention as next-generation catalysts. This work reviewed advances in the design and application of each common MOF type for biodiesel synthesis through esterification and transesterification process. MOF composites, MOF derivatives, and MOF composite materials exhibit superior catalytic performance and recyclability compared to pristine MOFs, making them highly recommended for future research and applications. Beyond summarizing yields and reaction conditions, we highlight mechanistic insights, stability issues, and catalysis performance. Special attention is given to functionalized and composite MOFs, bifunctional systems, and enzyme-MOF hybrids, which demonstrate superior performance compared to pristine MOFs. While UiO- and ZIF-based MOFs dominate current research, emerging systems such as Ca- and Cu-MOFs remain underexplored yet promising. We analyze the key features required in MOF materials for efficient biodiesel production and provide a comprehensive review and categorization of recent advancements. By contrasting MOFs with conventional heterogeneous catalysts and positioning this review against existing literature, we provide a comprehensive and critical perspective on the opportunities and challenges of MOFs in biodiesel catalysis.

## Abstract 2

Global energy demand and environmental concerns have intensified the search for renewable fuels, with biodiesel emerging as a sustainable substitute for petroleum diesel.

## Graphical abstract

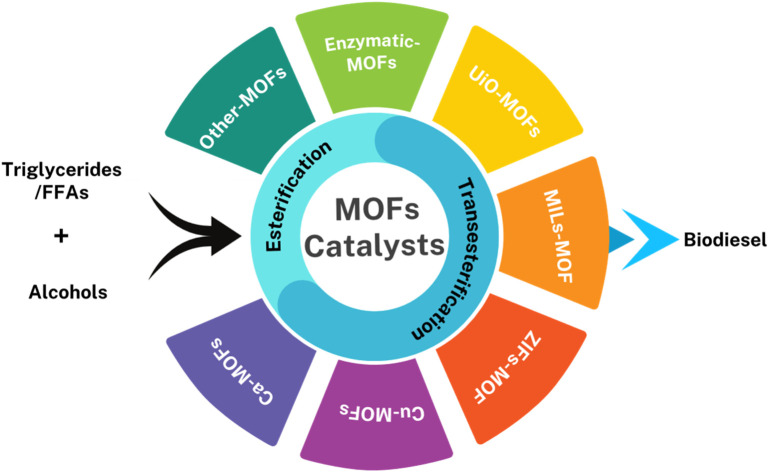

## 1 Introduction

The global energy crisis stems from over-reliance on finite fossil fuels such as coal, oil, and natural gas, which account for most energy consumption.^1–3^ Their extraction and combustion contribute significantly to greenhouse gas emissions, driving climate change through rising temperatures, extreme weather events, and biodiversity loss.^4–6^ Increasing scarcity, price volatility, and supply disruptions further threaten economic stability.^6–11^ To mitigate these challenges, transitioning to renewable energy sources, including solar, wind, and biofuels, is essential, offering sustainable alternatives with lower environmental impacts.^12–18^

Biofuels, derived from organic matter such as plant biomass, algae, or agricultural residues, provide renewable alternatives to fossil fuels, mitigating greenhouse gas emissions and reducing reliance on finite resources.^19–23^ Among these, biodiesel produced *via* transesterification of vegetable oils, animal fats, or waste cooking oils is biodegradable, compatible with conventional diesel engines, and amenable to existing infrastructure.^24–27^ Second-generation biofuels, derived from non-edible feedstocks such as agricultural residues, address food security concerns and reduce lifecycle carbon emissions by up to 78%.^28,29^ They also enhance energy security by reducing dependence on geopolitically sensitive fossil fuel reserves and support rural economies through agricultural employment and production.^26^

Conventional biodiesel production relies heavily on homogeneous catalysts like NaOH or KOH, which present drawbacks including complex separation, hazardous waste generation, and increased production costs.^30^ Scaling up advanced biofuels from algae or waste oils is also limited by technological and economic barriers. To overcome these challenges, innovative catalyst design, including heterogeneous and enzymatic systems, is essential for improving efficiency, lowering waste, and ensuring economic and environmental sustainability.^31–33^

The potential for the 2025 Nobel Prize in Chemistry^34^ to be awarded to pioneers (Kitagawa, Robson, and Yaghi) in the field of Metal–Organic Frameworks (MOFs) is a topic of interest given the significant advancements and applications of MOFs in recent years.^35–41^ MOFs have diverse applications such as water treatment,^42–48^ biomedical drug delivery,^49^ and gas separation.^50–54^ In particular, MOFs have emerged as promising heterogeneous catalysts for biodiesel production due to their unique features.^55–59^ Compared to conventional porous materials like carbon-based compounds or zeolites, MOFs^60–63^ offer superior chemical flexibility, versatile topologies, and adaptable synthesis conditions, allowing precise tailoring for diverse catalytic processes.^64–67^ For example, CuBTC MOF accomplished a 78.6% biodiesel yield under optimal conditions, retaining 76.6% activity after the first reuse cycle.^68^ Similarly, CaO–ZrO_2_ MOF-derived catalysts reached 97.12% yield, maintaining stability over five cycles.^69^ Structural designs like UiO-66@HAp, synthesized *via* impregnation, attained 97% yield owing to uniform pore structures, while microwave-assisted Fe(iii)-substituted MOFs reached 99.14% yield. MOFs demonstrate superior thermal stability; UiO-66@HAp showed no structural degradation after multiple uses, as confirmed by thermogravimetric analysis.^70^ Their reusability and robustness, unlike homogeneous catalysts, make MOFs cost-effective and sustainable for industrial-scale biodiesel production. In summary, MOFs are innovative catalysts that play an imperative role in developing the efficiency and sustainability of biodiesel synthesis through esterification and transesterification.

**Fig. 1:**
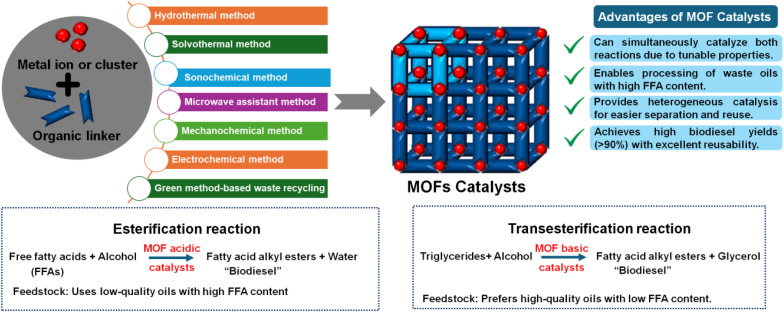
Demonstrates the synthesis approaches for MOF catalysts and their applications in esterification/transesterification processes for biodiesel synthesis.

Structurally, MOFs consist of inorganic metal nodes coordinated to organic ligands and can be synthesized *via* solvothermal, hydrothermal, or other techniques to achieve high crystallinity, surface area, and porosity (Fig. 1).^71,72^ Functionalization strategies such as Fe(iii) incorporation,^73^ cerium or chromium doping,^74^ or polyoxometalate encapsulation enhance active site availability and catalytic performance, achieving biodiesel yields exceeding 97–99%.^69^ MOFs' robustness, thermal stability, and reusability distinguish them from homogeneous catalysts, making them more cost-effective and sustainable for industrial biodiesel production.^30,70^

Recent years have witnessed a surge in MOF research for biodiesel catalysis.^66,75–78^ In continuation of previous research, this review aims to critically evaluate post-2020 advancements in MOF-based heterogeneous catalysts (*e.g.*, UiO-, MIL-, ZIF-, Ca-, and Cu-types) and their composites for biodiesel production *via* esterification/transesterification, emphasizing operational efficiency, mechanistic insights into reaction pathways, stability/reusability, functionalization strategies, and optimization for high yields from diverse feedstocks like waste oils and fatty acids. By contrasting MOFs with conventional heterogeneous catalysts, addressing unresolved challenges such as scalability and long-term stability, and contextualizing their potential to revolutionize biodiesel synthesis through unique physicochemical properties and alignment with circular economy principles, this work provides a structured roadmap for sustainable innovations, advancing beyond prior reviews through deeper analyses of underexplored systems and prospects.

## 2 Biodiesel production and its importance

Biodiesel is a renewable, biodegradable, and non-toxic fuel produced through transesterification/or esterification. This chemical process converts vegetable oils, animal fats, or waste cooking oils into a sustainable alternative to petroleum-based diesel.^79–81^ Its importance stems from its ability to reduce greenhouse gas emissions, lower sulfur content, and decrease particulate matter in exhaust, aligning with its carbon–neutral profile and environmental benefits.^50,54,82,83^ Compatible with existing diesel engines, used directly or blended with conventional diesel without requiring major modifications.^84^ Biodiesel diminishes reliance on non-renewable fossil fuels while maintaining infrastructure utility. Beyond ecological advantages, it bolsters agricultural and economic sectors by creating crops and waste product markets, thereby enhancing energy security, supporting sustainable development, and addressing climate change challenges through a cleaner, renewable energy solution.^85^

**Fig. 2:**
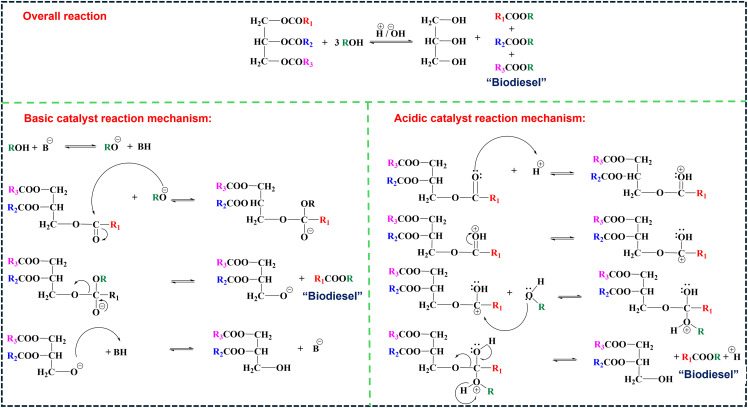
Schematic illustration of the transesterification reaction mechanism for biodiesel synthesis using homogeneous acid and base catalysts.

Biodiesel production primarily occurs through transesterification, a reaction where TGAs (TGAs) in oils or fats react with alcohol (typically methanol or ethanol) in the presence of a catalyst, yielding fatty acid methyl esters (FAME, biodiesel) and glycerol (Gly) (Fig. 1).^86–90^ For feedstocks with high FFA content, a two-step process is employed: esterification using an acid catalyst like sulfuric acid (H_2_SO_4_), first converts FAA to biodiesel, followed by transesterification, often with a base catalyst like NaOH or KOH to convert the remaining TGAs to biodiesel.^91,92^ Notably, homogeneous base catalysts achieve faster transesterification under mild conditions but are sensitive to FFA content. In contrast, homogeneous acid catalysts tolerate FAA but require higher temperatures and methanol ratios,^84,93–95^ as illustrated in Fig. 2. Common feedstocks for biodiesel production include edible oils (*e.g.*, palm, soybean, rapeseed), non-edible oils (*e.g.*, jatropha, karanja), waste cooking oil, and animal fats, with oleic acid (OA), rich sources like sunflower and peanut oils also utilized for their favorable fatty acid profiles.^96,97^

NaOH, KOH, or H_2_SO_4_ as homogeneous catalysts are widely used due to their high reactivity, low cost, and ability to accelerate reaction rates by lowering activation energy, ensuring high conversion efficiency in industrial settings.^98,99^ However, these catalysts dissolve uniformly in the reaction mixture, leading to significant drawbacks. Their inability to be easily separated from the product necessitates energy-intensive purification steps, generates waste streams, and increases production costs. While effective for transesterification, homogeneous base catalysts react with FAA to form soap, degrading product quality and complicating Gly separation. Acid catalysts, though suitable for esterification of FAA, can corrode equipment. Additionally, homogeneous catalysts cannot be reused, exacerbating environmental and economic challenges. These limitations highlight the trade-offs between their operational efficiency and the practical, financial, and sustainability hurdles they introduce in large-scale biodiesel production.^98,100,101^

Despite the effectiveness of homogeneous catalysts, their limitations, including separation, corrosiveness, and waste generation, have spurred a shift toward heterogeneous catalysts, which offer advantages such as ease of recovery, reusability, and reduced soap formation. Heterogeneous catalysts like solid metal oxides and MOFs remain insoluble during reactions, enabling sustainable and cost-effective biodiesel production through simplified recycling and lower environmental impact. MOFs, in particular, have appeared as a transformative innovation due to their capablity.^102–105^ These crystalline materials can integrate diverse metal ions (*e.g.*, Cu, Ca) and organic linkers, allowing precise control over their catalytic properties, such as incorporating acidic or basic sites for optimized esterification and transesterification. For instance, studies on Cu- and Ca-based MOFs demonstrate their efficiency in converting waste cooking oil into biodiesel, achieving yields up to 84.50% under optimized conditions.^106^ Additionally, MOFs operate effectively under mild reaction conditions and exhibit robustness in supercritical transesterification, reducing energy demands and enhancing scalability.^25,52,107,108^ Their adaptability to varying feedstock quality further underscores their potential to align biodiesel production with global sustainability goals, combining economic viability with environmental compatibility through reduced waste, energy consumption, and operational costs.^109^

## 3 Real-feed performance

Laboratory studies commonly employ OA or palmitic acid (PA) as model substrates to evaluate catalyst activity and kinetics. These controlled systems are valuable for probing mechanistic pathways of esterification or transesterification and for quantifying intrinsic reaction rates. However, OA/PA models lack the impurities and heterogeneity characteristic of industrial lipid streams, including water, FAA, phospholipids, metal ions, and oxidized compounds, all of which strongly influence catalyst deactivation and phase equilibria.^110,111^ As a result, performance in model systems cannot be directly extrapolated to real feedstocks. In contrast, real-feed studies utilizing waste cooking oil (WCO), microalgal oil, or wet lipid feeds present far more demanding reaction environments. These feeds often contain 5–40 wt% FAA and >5 wt% water, leading to soap formation, emulsification, and severe leaching or poisoning of active sites.^88,112,113^ Only a subset of catalysts has demonstrated genuine performance under such non-ideal conditions, maintaining activity and selectivity in the presence of impurities. For example, Heteropolyacid (HPA)–supported solids such as Cs_2.5_H_0.5_PW_12_O_40_/SiO_2_ or HPA–ZrO_2_ hybrids retain Brønsted acidity and structural integrity under humid conditions, achieving >90% ester yield from high-FFA WCO.^88^ Sulfonated carbon catalysts (*e.g.*, sulfonated biochar, carbonized lignin–SO_3_H) exhibit hydrophobic surfaces that repel moisture and sustain activity in wet WCO or microalgal oils, with turnover frequencies comparable to model OA reactions.^114,115^ MOF-derived oxides and sulfated zirconia–MOF composites possess dual acid-base sites, enabling simultaneous esterification of FAA and transesterification of TGAs. These systems show >95% conversion in microalgal oils containing both lipids and proteins.^93^ CaO-based bifunctional catalysts, when doped with Mg^2+^, Zn^2+^, or La^3+^, exhibit enhanced structural stability and moderate moisture tolerance, though still prone to leaching at water contents >2 wt%.^116^ Immobilized lipases (*e.g.*, Candida antarctica lipase B on hydrophobic supports) are slower but remarkably impurity-tolerant, achieving high conversions in wet and FFA-rich feedstocks with minimal by-product formation.^117^ Consequently, distinguishing model-feed (OA/PA) performance from real-feed (WCO/microalgal/wet) validation is essential for assessing industrial viability. Catalysts that perform robustly in impurity-rich feeds are the most relevant indicators of scalable biodiesel technology.

## 4 Structure–activity relationships in MOF-based catalysts for biodiesel production

The catalytic performance of MOFs in biodiesel synthesis depends strongly on their structural chemistry (Table 1). Parameters such as metal node composition, linker functionality, porosity, hydrophobicity, and acid/base balance govern the reactivity, stability, and reusability of MOFs in both esterification of FAA and transesterification of TGAs. Understanding how these features interact with feed characteristics ranging from model fatty acids (*e.g.*, oleic acid) to real oils (*e.g.*, WCO, microalgal oil) is crucial for translating laboratory activity into practical, industrial relevance.

**Table 1:** Summarized structure–activity relationships in MOF-based catalysts for biodiesel production

MOF system	Active sites	Structural strengths	Limitations	Feed stock	Ref.
UiO-66(Zr), UiO-66-SO_3_H	Strong Brønsted/Lewis acid, hydrolytically stable	Robust Zr_6_ nodes; tunable acidity; hydrophobic modification possible	Microporous, diffusion limits for TAGs	FAA/high-acid WCO	120
MIL-100(Fe), HPA@MIL-100	Dual acid sites; mesoporous	Large cages, easy guest acid loading, good reuse	Less hydrophobic, mild Fe leaching	Mixed FFA + TAGs oils	11
HKUST-1 (Cu-BTC)	Strong Brønsted/Lewis	High initial activity, low-T operation	Hydrolytic instability	Dry FFA models	121
ZIF-8/Hierarchical ZIF	Basic & hydrophobic	Stable, good for bulky TAGs & as carrier	Micropore diffusion limits unless hierarchical	TAGs oils, WCO	125
Ca-MOF/CaO@ZnO	Strong base	High basicity, improved moisture tolerance	Limited for high-FFA feeds (needs acid step)	Neutral/low-acid oils	11

### 4.1 Metal node chemistry and hydrothermal stability

The choice of metal node largely dictates the framework's Lewis/Brønsted acidity and its hydrolytic resistance under wet reaction conditions. UiO-66 feature robust Zr_6_O_4_(OH)_4_ clusters that tolerate alcohol/water environments and provide coordinatively unsaturated Lewis sites. These attributes enable UiO-66 to sustain high activity in oleic-acid esterification even under moisture, outperforming less stable Cu-carboxylate frameworks.^118–120^ Fe-based MIL-100(Fe) combines Lewis-acid Fe^3+^ centers with µ_3_-oxo bridges that introduce mild Brønsted acidity. Such dual functionality allows efficient conversion of high-FFA oils, especially when heteropolyacids (HPAs) are encapsulated within their large cages to reinforce acidity and water tolerance.^31^ Cu-based HKUST-1, while highly active due to exposed Cu^2+^ Lewis sites, exhibits poor hydrolytic stability; Cu–carboxylate bonds hydrolyze readily in methanol/water mixtures, leading to structural collapse in wet or impure feeds.^121^ ZIF-8 provide moderate basicity and zeolite-like robustness derived from strong Zn–N coordination, showing superior durability during TAGs transesterification and as carriers for enzymes or nanoparticles.^122^ Ca-MOFs and their thermally derived composites (*e.g.*, CaO@ZnO) deliver high basicity suited for TAGs transesterification. MOF-mediated synthesis stabilizes CaO nanophases, enhancing moisture tolerance and preventing deactivation common in bulk CaO catalysts.^11^

### 4.2 Linker functionality and tunable acidity

Linker modification enables fine control of Brønsted acidity and hydrophilicity. Sulfonated UiO-66 (UiO-66-SO_3_H) and amine-functionalized variants display significantly higher rates in oleic-acid esterification due to enhanced proton-donor capacity.^120^ In MIL-100(Fe), encapsulating Polyoxometalate (POM) Sulfonic acid functionalized ionic liquid provides additional strong acid sites while maintaining framework integrity, yielding >95% conversion of FAA in high-acid waste oils.^123^ Defect-rich HKUST-1 synthesized under solvent-free conditions exposes additional Cu^2+^ sites, improving conversion in model FAA but still sensitive to water ingress.^121^ Such tunability underscores how the acid strength and distribution within MOFs can be matched to the acidity and water content of specific feeds.

### 4.3 Porosity and diffusion effects

Esterification of FAA proceeds with relatively small molecules, whereas TAGs transesterification involves bulky substrates; thus, pore size and hierarchy become key design levers. The microporous UiO-66 (∼0.6 nm windows) provides sufficient accessibility for small FAA but suffers from diffusion limitations in oils unless defects or mesoporosity are introduced.^119^ MIL-100(Fe), possessing large cages (2–3 nm) and interconnected mesopores, readily accommodates TAGs molecules and immobilized acids, facilitating simultaneous esterification/transesterification.^31^ Hierarchical ZIF-8 or composite ZIFs (*e.g.*, ZIF-11@ZIF-8) reduce diffusion resistance and improve conversions of complex triglyceride feeds while maintaining high surface area.^124^

### 4.4 Hydrophobicity and water tolerance

Hydrophobic surfaces mitigate deactivation caused by water accumulation and soap formation in FFA-rich or wet feeds. Hydrophobized UiO-66(Zr) synthesized under solvent-free conditions exhibits higher methyl-oleate yields at ambient temperature due to reduced water adsorption and favorable alcohol–oil interfacial behavior.^122^ ZIF-8's inherent hydrophobicity enhances phase compatibility with oils, supporting better mass transfer and stability in wet WCO systems or as enzyme supports (*e.g.*, lipase@ZIF-8).^125^ Conversely, HKUST-1 lacks hydrophobicity, explaining its poor retention of activity in moist environments.^126,127^ Hydrophobicity therefore acts synergistically with acid/base sites to maintain active-site accessibility and equilibrium conversion.

### 4.5 Acid/base balance and feed complexity

For pure FAA (*e.g.*, OA), strong Brønsted acidity is sufficient; for mixed or TAGs-rich feeds, bifunctional catalysts are necessary. UiO-66-SO_3_H and HPA@MIL-100(Fe) exemplify strong acid catalysts ideal for FFA esterification. Ca-MOF-derived CaO@ZnO or ZIF-8 composites offer the basic sites required for TAG transesterification, while maintaining stability against water and CO_2_ poisoning.^11^ Integrated systems combining acid and base sites (or sequential pretreatment) achieve high conversions of high-FFA WCO or microalgal oils without external neutralization steps.^128^ Finally, these correlations indicate that Zr- and Fe-based acidic frameworks exhibit superior efficiency in FFA esterification, whereas Ca- and Zn-based basic or bifunctional frameworks are better suited for TAGs transesterification. The integration of hydrophobic surface properties, hierarchical mesoporosity, and balanced acid-base functionality remains the most effective design strategy for narrowing the performance gap between model substrates and real feedstocks.

## 5 Contributions of Brønsted and Lewis acid-base sites in MOF-catalyzed biodiesel reactions

The catalytic performance of MOFs in biodiesel synthesis arises from the interplay between Brønsted acidity, Lewis acidity, and basic functionality. These distinct active sites govern different mechanistic pathways depending on the feedstock type FAA *versus* triglyceride-rich oils and determine how efficiently the framework sustains conversion in the presence of water, glycerides, and impurities.

### 5.1 Brønsted *vs.* Lewis acid pathways in FFA esterification

Esterification of FAA such as oleic acid follows a classical acid-catalyzed mechanism involving carbonyl activation and proton transfer. Here, Brønsted acid sites (–SO_3_H, µ_3_–OH, or encapsulated HPA species) protonate the carboxyl oxygen, lowering the LUMO energy of the carbonyl and facilitating nucleophilic attack by methanol. Pyridine-adsorbed FT-IR spectra (Py-IR) consistently reveal the dominance of Brønsted acidity through characteristic bands at ∼1540 cm^−1^ (PyH^+^), as reported for UiO-66-SO_3_H and HPA@MIL-100(Fe) systems.^118,129^ NH_3_-TPD profiles show broad desorption peaks above 300 °C, confirming strong protonic acid strength.^110^ Periodic DFT simulations on defective UiO-66 reveal that missing linkers generate adjacent Zr–OH–Zr motifs, which act as cooperative Brønsted–Lewis pairs; the µ_3_–OH donates a proton while neighboring Zr^4+^ stabilizes the transition state, reducing the activation barrier for FFA esterification by ∼25 kJ mol^−1^.^130^ Brønsted-rich frameworks, therefore, excel in FFA esterification because they tolerate water (protons regenerate after each turnover) and maintain structural integrity under polar reaction media. UiO-66-SO_3_H, MIL-100(Fe) with encapsulated HPA, and sulfonated carbon@MOF hybrids exemplify this class of efficient acid catalysts. In contrast, Lewis acid sites, typically unsaturated metal centers (Zr^4+^, Fe^3+^, Cu^2+^) activate the carbonyl group *via* coordination rather than protonation. Py-IR spectra show bands at ∼1450 cm^−1^, diagnostic of Lewis acid–pyridine complexes.^131^ In defective UiO-66 and MIL-100(Fe), these sites complement Brønsted acidity by polarizing carbonyls and stabilizing alkoxy intermediates. DFT charge-density maps confirm charge depletion on carbonyl oxygen upon Zr coordination, explaining the synergy between both acid types.

### 5.2 Lewis and basic sites in transesterification

Transesterification of triglycerides proceeds through a different route that requires alcohol deprotonation and methoxide generation processes favored by Lewis basic or amphoteric sites rather than strong acids. CO_2_-TPD measurements identify medium and strong base sites desorbing between 250–450 °C, which correlate with high triglyceride conversion rates.^88,114^ XPS spectra (O 1s ≈ 530 eV, N 1s ≈ 399 eV) confirms the presence of oxide and imidazolate anion responsible for alcohol activation. In ZIF-8, the Zn–N framework provides mild basicity, suitable for forming surface methoxide species (Zn–OCH_3_) without excessive soap formation. DFT studies show that the imidazolate linker's lone pair interacts with methanol, lowering the deprotonation energy by ∼0.3 eV compared with pure ZnO.^65^ In Ca-MOF-derived CaO@ZnO composites, CO_2_-TPD and XPS reveal strong O^2−^ sites stabilized by ZnO interfaces, yielding rapid transesterification of soybean and waste oils even at room temperature.^11^ These findings align with mechanistic requirements: the base site must abstract a proton from methanol and then facilitate nucleophilic attack on the TAG carbonyl carbon. Certain amphoteric MOFs, such as MIL-100(Fe) or UiO-66 with defects, display dual acid-base functionality. NH_3_- and CO_2_-TPD analyses show overlapping desorption domains, indicating coexistent acidic and basic sites that can process mixed FFA + TAGs feeds without pretreatment.^132,133^ Brønsted acid sites efficiently catalyze FFA esterification and resist water, while Lewis and basic sites favor TAG transesterification but are moisture sensitive. Bifunctional catalysts like HPA@MIL-100–CaO and UiO-66–CaO balance both functions, preventing soap formation and sustaining high activity in wet WCO feeds.^134,135^ Additionally, hydrophobic surface engineering as shown for UiO-66 and ZIF-8 reduces surface hydroxylation and ensures stable conversion under aqueous or impure conditions.^136^ In summary, the convergence of spectroscopic evidence and DFT modeling underscores that MOF design for biodiesel catalysis must move beyond surface area optimization toward precise acid–base engineering. Defect modulation, post-synthetic functionalization (–SO_3_H, amines), and heterojunction formation (MOF@metal oxide) are promising avenues to tailor site distribution and hydrophobic balance. Future studies should report quantitative site densities (µmol g^−1^) from Py-IR and NH_3_/CO_2_-TPD, correlated directly with kinetic constants for both reactions under standardized FFA and WCO feeds.

## 6 Progress on MOF catalysts for biodiesel production

Advances in MOF synthesis and functionalization have unlocked new possibilities for their application in biodiesel synthesis.^108,137^ The ability of MOFs to serve as many active sites in a confined area is one of their main advantages in catalysis. This characteristic has been leveraged to develop MOF-based catalysts with superior catalytic performance. For instance, introducing acidic or basic functional groups into MOFs significantly enhances their catalytic activity for transesterification reactions. The esterification of OA with methanol has shown high catalytic efficiency and reusability in acid-functionalized MOFs like MIL-101(Cr)–SO_3_H, highlighting their potential for the synthesis of biodiesel.^138^

Furthermore, MOFs can be engineered to possess both Lewis and Brønsted acid sites, further enhancing their catalytic efficiency. In the transesterification of TGAs, bifunctional MOFs like UiO-66-NH_2_-SO_3_H have demonstrated exceptional performance, producing high yields of biodiesel under mild reaction conditions.^118^ This dual functionality facilitates the simultaneous activation of both methanol and triglyceride molecules, thereby promoting a more efficient catalytic process. Magnetic materials are pivotal in diverse scientific applications, enabling facile separation and recovery.^139–148^ Catalysts based on magnetic materials are important because their magnetic properties enable easy separation and recovery of the catalyst using an external magnetic field.^146,147,149^ El-Nahas *et al.* found that functionalized cellulose-magnetite nanocomposites can act as catalysts in the esterification of OA with methanol. To esterify OA with methanol, Wang *et al.* used tin(ii) chloride as a catalyst.^150^ In order to create fatty acid ethyl esters (FAEEs), Caratelli *et al.* estimated the efficacy of UiO-66(Zr) and UiO-66(Zr)–(COOH)_2_ in the esterification process of levulinic acid with ethanol.^151^ D. Costa *et al.* prepared UiO-66(Zr) and UiO-66(Zr)–NH_2_ utilizing a solvothermal method, achieving improved esterification performance.^152^ The enhanced catalytic performance of this system was facilitated by its dual activation mechanism, which involved the zirconium centre acting as a Lewis acid and the amino group acting as a Brønsted base. Li *et al.* utilized this modified catalyst to esterify OA and create biodiesel by integrating *p*-Toluenesulfonic acid (PTSA) into UiO-66(Zr) while preserving its original framework.^153^

Another critical area of research focuses on the stability and reusability of MOF catalysts. Heterogeneous MOF catalysts are readily separated from the reaction mixture and can be used frequently without a noticeable decrease in activity. For example, Zr-based MOFs such as UiO-66 have demonstrated remarkable stability and reusability in biodiesel production, maintaining consistent catalytic performance across multiple reaction cycles.^154,155^ Additionally, the development of continuous-flow processes using MOF catalysts has demonstrated significant promise for raising the production of biodiesel's scalability and efficiency.^156^ M. Saidi *et al.* successfully implemented a continuous-flow process for biodiesel synthesis using a MOF catalyst, achieving high yields and productivity with minimal catalyst degradation.^108^

**Fig. 3:**
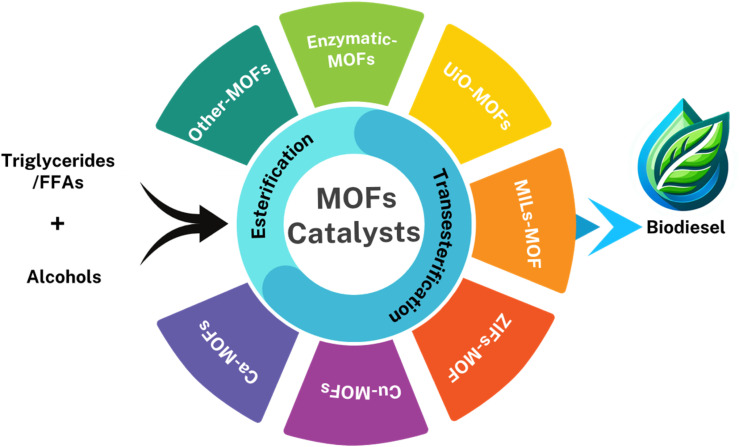
Schematic representation of diverse MOFs employed in biodiesel production.

In conclusion, MOFs have become sophisticated catalysts to produce biodiesel, leveraging their unique characteristics. To further improve MOFs' catalytic performance and stability and open the door to more ecological and effective biodiesel synthesis methods, ongoing research attempts to optimize MOF structures and create new functionalization. In the current study, we reviewed and sorted out the recent progress on the most common MOFs (Fig. 3) used in this area of research to highlight their drawbacks and simplify the roadmap for efficient biodiesel production in the future. Also, this review systematically analyzes the optimization of MOF catalysts for high-yield biodiesel production, offering a roadmap to enhance catalytic efficiency and scalability.

### 6.1 UiO-MOF for biodiesel production

UiO-MOFs (Universitetet i Oslo) are highly stable and porous materials composed of metal clusters interconnected by organic linkers, which have garnered significant attention in catalysis and sustainable energy applications.^157^ In the realm of biodiesel synthesis, UiO-MOFs serve as efficient heterogeneous catalysts due to their tunable porosity, high surface area, and robust chemical stability, which facilitate the transesterification of TGAs and esterification of FAA.^158,159^ Their structural versatility allows for functionalization with active sites, enhancing catalytic performance and selectivity. In this literature review, we have comprehensively summarized the recently published work on the synthesis, characterization, mechanisms, and application of UiO-MOFs in biodiesel production, highlighting their potential to address challenges such as catalyst recyclability, reaction efficiency, and environmental sustainability.^160,161^ This section aims to provide a consolidated resource for researchers exploring UiO-MOF materials for renewable energy solutions. Table 2 review the various UiO-MOF catalysts explored for biodiesel production, detailing the feedstocks and yields.

**Table 2:** Summary of recent progress on using UiO-MOFs for biodiesel production

UiO-MOFs catalyst	Feedstock	Alcohol : oil molar	Catalyst loading	Temp (°C)	Time (h)	Reactor mode	Biodiesel yield (%)	Cycles	Acid/Base site density	Ref.
MTV-UiO-66	Butyric acid	2 : 1	1 wt%	110	24	Batch (reflux)	92.00	4	Defects ∼1.9 (per Zr) (no added acid sites)	170
PTSA/UiO-66	Palmitic acid	8 : 1	≈9.7 wt%	100	4	Batch (sealed)	97.00	9	∼1.36 mmol H^+^ g^−1^ (0.26 g PTSA g^−1^)	171
MSA/UiO-66	Palmitic acid	8 : 1	≈9.4 wt%	100	4	Batch	97.00	5	∼2.57 mmol H^+^ g^−1^ (0.247 g MSA g^−1^)	171
NiHSiW/UiO-66	OA	—	—	—	—	Batch	86.70	—	—	172
UiO-66(Zr)–NH_2_	OA	10 : 1	10 wt%	90	4	Batch	97.30	5	(Lewis acid Zr nodes)	132
UiO-66(Zr)– NO_2_	OA	10 : 1	10 wt%	90	4	Batch	90.70	5	(Lewis acid Zr nodes)	132
UiO-66(Zr)	OA	10 : 1	10 wt%	90	4	Batch	86.30	5	—	132
UiO-66/SA	OA	15 : 1	10 wt%	90	4	Batch (sealed)	94.50	4	2.28 mmol g^−1^ acid sites	159
[CH_2_(COOH)_2_IM] HSO_4_@H-UiO-66	OA	8 : 1	8 wt%	70	2	Batch (microwave)	93.82	5	Brønsted + Lewis acid sites (IL + Zr)	271
ZrSiW/UiO-66	OA	—	—	120	—	Batch (sealed)	98.00	5	1.74 mmol g^−1^ acidity	171
UiO-66-NH_2_@MnFe_2_O_4_	Edible oil	20 : 1	10 wt%	70	6	Microwave batch	97.81	7	(Bifunctional acid/base)	158
BDC@HSiW@UiO-66	OA	30 : 1	∼5 wt%	130	4	Batch (autoclave)	81.50	6	Strong Brønsted acidity (NH_3_-TPD)	160
UiO-66@HAp	Palm oil	6 : 1	6 wt% (of oil)	70	1	Batch (microwave)	97.00	5	Basic sites (CaO in HAp, not quantified)	70
UiO-66/SFN	OA	15 : 1	5 wt% (est.)	100	4	Batch	96.20	5	(Brønsted acid sulfate on Zr)	161

Chaemchuen and colleagues created the UiO-66 catalyst to esterify OA with methanol.^162^ With an *E*_a_ of 54.9 ± 1.8 kJ mol^−1^, kinetic analysis demonstrated that the sorption of methyl oleate was irreversible. Furthermore, Wei's research team created flawed UiO-66 catalysts, especially for the ethanol-levulinic acid esterification process.^163^ Their findings highlighted that hydroxyl groups and unsaturated Zr_6_ nodes significantly enhanced the catalytic performance. Remarkably, after five cycles, the defective UiO-66 catalyst retained 75% of its initial activity, demonstrating excellent stability over multiple catalytic cycles. The structural integrity and active site characteristics are analyzed using X-ray Photoelectron Spectroscopy (XPS). To further investigate the interaction between SO_4_ and Zr, the composition elements of UiO-66/SFN and UiO-66/SSN are analyzed, comparing the Zr 3d and S 2p spectra through high-resolution XPS patterns. It is revealed that the increase in Zr 3d binding energy for UiO-66/SSN indicates a reduction in electron density on Zr due to sulphate interaction, which enhances the Lewis acid sites. The slight decrease in S 2p binding energy, which suggests a modified electronic environment of sulphate influenced by zirconium, further supports this interaction.

H. Li *et al.*^161^ suggested a novel synthesis method for advancing and effective heterogeneous acid catalyst for biodiesel synthesis using UiO-66 and ammonium sulphate ((NH_4_)_2_SO_4_) in order to address catalytic stability issues that arose during successive batch trials. UiO-66 was added after ammonium sulphate was dissolved in DI water to create a 0.1 mol L^−1^ solution. After an hour of stirring, the mixture was dried for six hours at 120 °C. The catalyst precursor was the dried solid known as UiO-66/S-P. The first calcined catalysts, UiO-66/SFA and UiO-66/SFN, respectively, were produced by calcining the catalyst for two hours at 500 °C in air and nitrogen atmospheres. UiO-66/SFN underwent secondary calcination in a nitrogen environment to further improve catalytic stability. At a rate of 10 °C per minute, the temperature was raised from room temperature to 500 °C and held there for two hours. The resulting solid was identified as UiO-66/SSN, the last catalyst. Although the catalysts' surface area and pore volume decreased after the second calcination, XPS analysis showed improved interactions between zirconium and sulfate. Enhanced electron attraction to sulfur significantly increased the Lewis and Brønsted acidity of UiO-66/SSN. UiO-66/SFN attained the highest esterification conversion rate of 96.20% under ideal catalyst preparation conditions. This was accomplished with a methanol-to-OA molar ratio of 8, an 8% catalyst loading, and a 2 h reaction temperature of 70 °C. The second calcination of UiO-66/SFN under a nitrogen atmosphere further minimized the conversion decline, reducing it by 66.25% over consecutive reaction cycles.

**Fig. 4:**
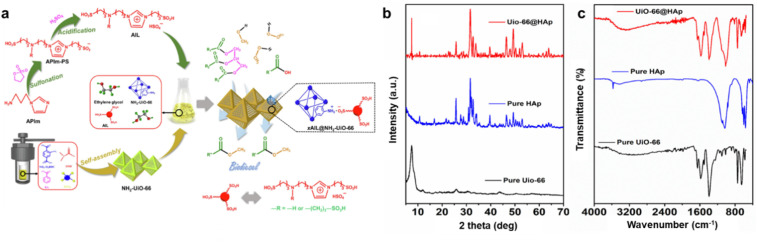
(a) Schematic of preparation for NH_2_-UiO-66 and its application in biodiesel synthesis. This figure has been adapted/reproduced from ref. 164 with permission from Elsevier Ltd, Copyright 2022. (b and c) XRD patterns and FTIR spectra of UiO-66, Hap, and its nanocomposite, respectively. This figure has been adapted/reproduced from ref. 70 with permission from Wiley-VCH, Copyright 2024.

Li *et al.* utilized a defect coordination method to support PTSA on UiO-66(Zr).^153^ Their findings showed that PTSA was well incorporated into UiO-66(Zr), and under ideal circumstances, OA conversion to biodiesel peaked at 91.30%. However, after four cycles, the conversion rate dramatically decreased to 76.65% during the reusability analysis, which was consistent with the catalyst's observed elimination of Zr and S elements. Lu *et al.* incorporated acidic ionic liquids (AILs) into the NH_2_-UiO-66 framework using an acid-base interaction approach.^164^ This system accomplished a conversion rate of 95.22% for OA after 6 hours of esterification. Even after six cycles, the conversion remained at 90.42%, confirming the synergistic enhancement between the –NH_2_ group of NH_2_-UiO-66 and the – SO_3_H group of AILs. As shown in Fig. 4a, the schematic illustrates the preparation process for NH_2_-UiO-66, detailing the steps and reagents involved in its synthesis and showing potential in biodiesel production. A solid catalyst made of sulfonated ionic liquids (AILs) and HPW-modified UiO-66-2COOH was researched by Xie's research group for the one-pot trans/esterification of acidic vegetable oils.^165^ Even with a feedstock that contained 9% FAA and 3% water, the catalyst showed excellent performance, reaching a conversion rate of 95.80%. A mean pore size of 16.07 nm, a surface area of 8.63 m^2^ g^−1^, and a pore volume of 0.04 cm^3^ g^−1^ were determined by structural analysis.

In order to create different UiO-66-based solid superacids, W. Peng and associates quaternized UiO-66 with 1,3-propane sultone and switched ions with H_2_SO_4_ or HSO_3_CF_3_ (ref. 166) the resulting catalysts, UiO-66-[C_3_NH_2_][SO_4_H] and UiO-66-[C_3_NH_2_][SO_4_CF_3_] demonstrated biodiesel yields ranging from 86.60% to 98.40%, with UiO-66-[C_3_NH_2_][SO_3_CF_3_] showing superior performance compared to the others. By combining HPW and UiO-66-NH_2_, Yang *et al.* created a hybrid catalyst that offered both Lewis and Brønsted acid functionalities.^167^ This catalyst achieved 91.20% biodiesel yield from Lathyris L. oil esterification and retained its performance over four cycles without significant deactivation. The catalyst's high acid density (1.7 mmol g^−1^) and large surface area (301.60 m^2^ g^−1^) contributed to its efficiency. Further, ZrSiW/UiO-66 nano-hybrid MOFs were prepared for the esterification of OA.^73^ A maximum conversion rate of 98.00% was attained using 0.24 g of catalyst at 150 °C and a 1 : 20 molar proportion of OA to methanol. Furthermore, there would be no appreciable performance loss if the catalyst was reused up to six times. The PW_12_@UiO-66 composite was created by Z. Yuxin *et al.*^168^ using a two-step procedure that included the production of Zr_6_(OH_4_)O_4_(AC)_12_ and the low-temperature self-assembly of PW_12_@UiO-66. After ten recycling cycles, the compound retained its structural stability and catalytic activity while converting 90% of soybean oil to biodiesel.

In another investigation, the nanocomposite UiO-66@HAp was successfully synthesized by the authors using a straightforward impregnation method, and the catalyst was thoroughly characterized using a variety of techniques, such as FTIR and XRD.^70^ The distinctive peaks of UiO-66 and HAp were visible in the XRD spectra (Fig. 4b), indicating that the nanocomposite was successfully formed without materially changing the structure of UiO-66 or changing the crystallinity of HAp. This suggested that the components' distinct crystallographic qualities were preserved because the impregnation process did not significantly alter any of their structures. FTIR analysis confirmed the successful synthesis of UiO-66@HAp by identifying characteristic functional groups from both UiO-66 and hydroxyapatite (HAp). The presence of phosphate and hydroxyl groups indicated the effective incorporation of HAp into the MOF structure.

Additionally, the FTIR spectra (Fig. 4c) verified the composite's integrity, showcasing peaks corresponding to UiO-66's organic linkers and HAp's mineral components, highlighting its potential for catalytic applications. The study also investigated how different factors, including temperature, methanol-to-oil ratio (MTOR), catalyst loading, and reaction time, affected the amount of biodiesel produced. The findings demonstrated that 70 °C, 60 min, and an ideal MTOR of 6 : 1 were the ideal reaction conditions to produce biodiesel, resulting in a 97% biodiesel yield. Furthermore, it was found that the best catalyst loading for optimizing the synthesis of biodiesel was 6 wt%. These results demonstrate the UiO-66@HAp nanocomposite's effective use as a catalyst in the synthesis of biodiesel, attaining high conversion efficiency under perfect conditions.

**Fig. 5:**
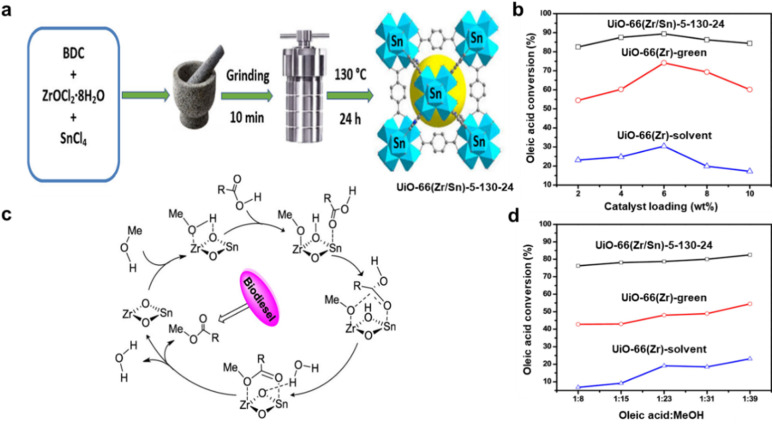
(a) The preparation of UiO-66(Zr/Sn^4+^)-5-130-24. (b and d) Catalyst loading and OA : MeOH molar ratio of three catalysts effect on OA conversion. (c) Reaction pathway including active sites for the esterification of OA over UiO-66(Zr/Sn)-5-130-2. This figure has been adapted/reproduced from ref. 169 with permission from American Chemical Society, Copyright 2023.

Finally, using a solvent-free method, Abou-Elyazed *et al.*^169^ synthesised UiO-66(Zr/Sn^4+^)-5-130-24 and converted OA to biodiesel with methanol at 333 K with a 98.80% conversion rate, compared to 86.2% for UiO-66(Zr). As illustrated in Fig. 5a, the schematic depiction comprises the process of preparing UiO-66(Zr/Sn^4+^)-5-130-24. Fig. 5b and d illustrates the effects of catalyst loading and the OA : MeOH molar ratio on OA conversion over UiO-66(Zr/Sn^4+^)-5-130-24, UiO-66-Zr-green, and UiO-66-Zr-solvent, respectively. As shown in Fig. 5b, increasing the catalyst loading from 2 to 6 wt% improved the biodiesel yield for all three catalysts. However, further increases led to a decrease in conversion, likely due to mass transfer limitations caused by the formation of viscous slurries. In Fig. 5d, UiO-66(Zr/Sn)-5-130-24 demonstrated the highest catalytic activity, nearly twice that of UiO-66(Zr)-green, highlighting the synergistic effect of the dual Lewis acid sites (Zr-site and Sn-site). Fig. 5c shows a potential reaction pathway for UiO-66(Zr/Sn)-5-130-24 based on the condition of its active sites. To create the tetrahedral intermediates (carbocation), the carbonyl group of OA is first adsorbed on the active sites of Zr or Sn in UiO-66(Zr/Sn)-5-130-24. The carbocation intermediates in the reaction medium were then attacked by deprotonated methanol molecules. Eventually, methyl oleate is yielded by the release of water molecules.

MTV-UiO-66 structures were studied for the synthesis of butyl butyrate.^170^ Two mixed-linker systems were used to synthesize MTV-UiO-66(COOH)_2_ and MTV-UiO-66(OH)_2_, resulting in the creation of nine different structures. Because functionalized linkers reduced pore volume and surface area, the final molar ratio values were marginally lower than the original values. Using a 1 wt% catalyst, the best results were achieved with MTV-UiO-66, which featured an initial molar composition of 25% BDC and 75% functionalized linkers. This catalyst used UiO-66(1A:3B) to achieve butyl-butyrate conversion rates of 89% and 92%, respectively. Notably, earlier research showed that highly defective single-component functionalized structures could achieve comparable outcomes with twice the catalyst quantity.

Zr-MOFs (UiO-66) modified with PTSA and methane sulfonic acid (MSA) were synthesized through a straightforward impregnation process. These catalysts incorporated supported sulfonic acids, specifically PTSA and MSA. These modified Zr-MOFs (PTSA/UiO-66 and MSA/UiO-66) were efficient heterogeneous acid catalysts for esterifying different alcohols (methanol and *n*-butanol) with FFTAs obtained from biomass (like palmitic and OA). Even after nine consecutive cycles, the catalytic activity of these UiO-66 materials functionalized with sulfonic acid, including MSA/UiO-66, remained strong. These results demonstrate the potential of MOFs supported by sulfonic acid, such as UiO-66, for the synthesis of biodiesel because of their superior reusability.^171^

NiHSiW/UiO-66 nanocatalysts were created and investigated for the production of biodiesel from methanol and OA.^172^ The large specific surface area, nano-sized catalyst, and synergistic interactions between the NiHSiW salts and the UiO-66 matrix were responsible for the optimized process's 86.70% conversion rate. This catalyst showed relative stability in producing biodiesel and good recyclability, retaining about 50% conversion after eight cycles. In order to produce biodiesel from OA through esterification, Abou-Elyazed *et al.* synthesised UiO-66(Zr)–NH_2_, UiO-66(Zr)–NO_2_, and UiO-66(Zr) materials.^132^ After four hours at 60 °C, they obtained high biodiesel conversion rates of 97.30%, 90.70%, and 86.30%, respectively. The order of catalytic performance was UiO-66(Zr)–NH_2_ > UiO-66(Zr)–NO_2_ > UiO-66(Zr). This is assigned to the introduction of NH_2_ groups in UiO-66(Zr), which enhances its catalytic activity compared to UiO-66(Zr) and UiO-66(Zr)–NO_2_. Added to, UiO-66(Zr)–NH_2_ has a higher *S*_BET_ surface area (823 m^2^ g^−1^) than UiO-66(Zr)–NO_2_ (649 m^2^ g^−1^), indicating better accessibility for reactants. After three cycles, these catalysts maintained a yield of about 60% biodiesel, demonstrating their good recyclability.

**Fig. 6:**
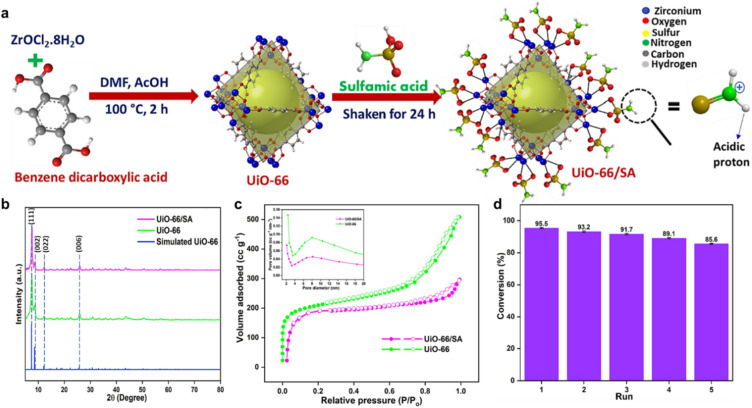
(a) Pathway of UiO-66/SA synthesis. (b) XRD spectra of UiO-66/SA, UiO-66, and simulated UiO-66. (c) N_2_-sorption/desorption isotherm of UiO-66/SA and UiO-66. (d) Recyclability performance of UiO-66/SA. This figure has been adapted/reproduced from ref. 159 with permission from Elsevier Ltd, Copyright 2024.

A new sulfamic acid-modified UiO-66 MOF (UiO-66/SA) catalyst was created to esterify OA and generate biodiesel.^159^ Under optimal conditions, a temperature of 65 °C, a molar ratio of 12 : 1 between methanol and OA, and a catalyst loading of 5 wt%, the catalyst demonstrated a high level of catalytic activity, yielding a biodiesel yield of 98.5%. Additionally, the UiO-66/SA catalyst demonstrated excellent reusability, maintaining significant activity after multiple cycles, which underscores its potential for sustainable biodiesel synthesis. Fig. 6a illustrates the stepwise synthesis route for UiO-66/SA. At the same time, the XRD spectra (Fig. 6b) showed regular crystallinity. The XRD pattern of UiO66/SA has a similar pattern to that of UiO-66, with distinctive peaks appearing at 2*θ* = 7.5°, 8.6°, 12.1°, and 25.7°. These peaks were well associated with the UiO-66 pattern, which corresponded to the planes (111), (002), (022), and (006), respectively. As demonstrated by the catalyst's type IV N_2_-adsorption/desorption isotherm with an H3-type hysteresis loop, the catalyst is mesoporous (Fig. 6c). The catalyst has *S*_BET_ of 561.2 m^2^ g^−1^, as opposed to 1009.9 m^2^ g^−1^ for pristine UiO-66. To assess reusability, the reaction was performed over five consecutive cycles under optimal conditions with a consistent recovery protocol (Fig. 6d), demonstrating sustained catalytic efficiency with >85% conversion retained after five cycles.

### 6.2 MILs-MOF for biodiesel production

A class of MOFs known as Materials of Institute Lavoisier (MIL) has been thoroughly researched for a variety of uses, including the manufacturing of biodiesel.^173^ These MOFs have a porous structure with large surface areas and adjustable pore sizes, made up of metal or metal clusters connected by organic linkers.^43,45,47,174^ Because of its exceptional qualities, including its high surface area, outstanding thermal stability, and noticeable acidity, MIL-100 has attracted a lot of interest in the production of biodiesel.^175^ Unsaturated metal sites, which act as active sites for transesterification reactions, are the main cause of MIL-100's high acidity.^46,176^ Notable is the potential of MIL-MOFs as heterogeneous catalysts to produce biodiesel.^117,177^ These materials are not only highly efficient but also recyclable and reusable, offering a sustainable and cost-effective solution for biodiesel synthesis. Moreover, their structural versatility allows for modifications to enhance catalytic performance, further solidifying their role in advancing biodiesel production technologies.

Due to alkaline catalysts being able to extract protons from alcohol and producing potent nucleophilic species that react with carbocation intermediates in oils or fatty acids, they are thought to be very effective for producing biodiesel. This section highlights the application of alkaline MIL-MOFs in biodiesel production. For example, the carbonization of MIL-100(Fe) produces a magnetic mesoporous material, Fe@C, which becomes an efficient heterogeneous basic catalyst when combined with strontium oxide (SrO), forming Fe@C–Sr. Even with up to 6 wt% free water present, this catalyst achieves a 91.93% conversion rate when transesterifying palm oil with methanol at a reaction temperature of 600 °C.^178^ Similarly, MM-SrO and ST-SrO catalysts are produced by calcining SrO-MIL-100(Fe), which is created by mechanical mixing and *in situ* titration of SrCO_3_ with MIL-100(Fe). At a catalyst amount of 8%, and a reaction temperature of 65 °C for 30 minutes, MM-SrO can produce a maximum biodiesel conversion rate of 96.19%. After three cycles, the conversion rate stays constant at 82.49%.^179^

Another notable alkaline catalyst, CAM750, integrates CaO into MIL-100(Fe) using calcium acetate. With a catalyst amount of 4%, this catalyst exhibits exceptional stability and magnetic properties, achieving a 95.07% conversion rate at 65 °C in 2 hours.^180^ Even though alkaline catalysts are typically more active, processing oils with a high content of FAA can cause saponification, which results in the formation of soap. Similarly, Xie and Wang synthesized CoFe_2_O_4_/MIL-88B(Fe)–NH_2_/(Py-Ps) PMo for production of biodiesel from soybean oil and FAA *via* one-pot transesterification-esterification, a 95.60% conversion was obtained with no discernible loss of its activity after five cycles.^181^

**Fig. 7:**
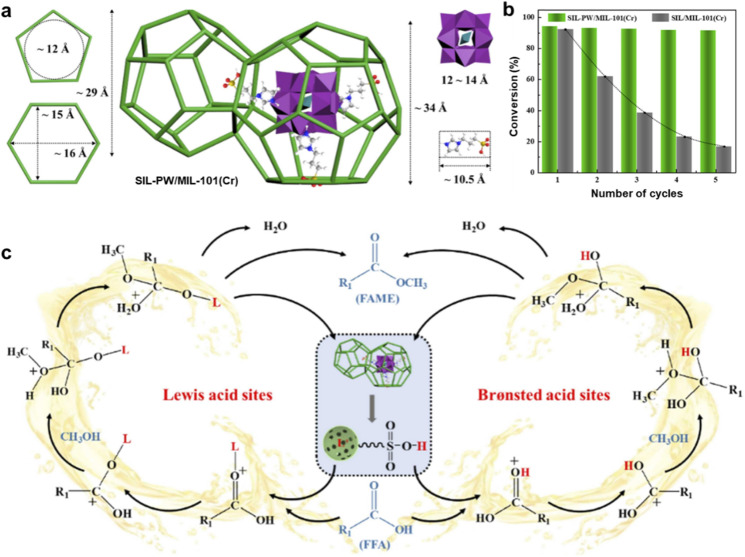
(a) Geometrical configuration of the SIL-PW/MIL-101(Cr) catalyst. (b) Reusability of SIL-PW/MIL-101(Cr) catalyst. (c) Esterification of FFA reaction pathway over the SIL-PW/MIL-101(Cr) catalyst. This figure has been adapted/reproduced from ref. 184 with permission from Elsevier Ltd, Copyright 2022.

Acid catalysts are frequently used to address this problem. They work especially well with oils that contain a lot of FAA. For instance, MIL-100(Fe), functionalized with sulfonic acid (MF-SO_3_H), functions as a heterogeneous acid catalyst that is both effective and recyclable. With an MTOR of 10 : 1 and an 8 wt% catalyst concentration, the catalyst enables the esterification of OA with methanol, reaching a maximum conversion of 95.86% at 70 °C in 2 hours. After five cycles, the conversion rate stays at 88.5%.^182^ A powerful catalyst for the esterification of OA with ethanol, P_3_W_12_O_40_@MIL-100, is created when polyoxometalate (POM) and MIL-100(Fe) are combined. In ideal situations (molar ratio of 11 : 1, 15 wt% catalyst, 111 °C, and 5 hours), the conversion rate reaches 94.6%.^123^ This catalyst benefits from the synergistic properties of ionic liquid, POM, and MOF, offering enhanced activity and stability. A very stable and effective catalyst was produced by synthesizing the CAM750 using MIL-100(Fe) and calcium acetate. The dominant active sites were identified as Ca_2_Fe_2_O_5_ and CaFe_3_O_5_. XRD patterns confirmed that the precursor retained a structure similar to MIL-100(Fe) and that calcium acetate was evenly distributed within the material.^180^ The MIL-101 catalyst has been extensively studied for its potential applications in various catalytic processes.^183^ A highly efficient heterogeneous catalyst, SIL-PW/MIL-101(Cr), was designed for biodiesel synthesis by integrating SO_3_H-functionalized ionic liquid (SIL) with MIL-101(Cr) using phosphotungstic acid (HPW) as a bridging agent. This catalyst achieved a remarkable 94.30% conversion of OA, showcasing its outstanding catalytic performance. XRD analysis (Fig. 7a) confirmed the preservation of the mesoporous structure of MIL-101(Cr) during the immobilization procedure, facilitating the diffusion of large-sized reactants. The catalyst exhibited excellent stability and reusability, maintaining 91.80% conversion efficiency after five cycles (Fig. 7b). The enhancement in the catalytic activity is attributed to the combined Brønsted acid sites from the SO_3_H groups and Lewis acid sites, which provide dual functionality that significantly boosts performance. Importantly, the catalyst's stability over multiple cycles can be linked to the stable presence of both acid sites (Fig. 7c). This study underscores the potential of using a bridging strategy to design heterogeneous catalysts with multiple active sites, offering a new approach for efficient and sustainable catalytic processes.^184^

The use of AIL/HPMo/MIL-100(Fe) as a catalyst for the production of biodiesel from acidic oils, such as soybean oil and FAA, through ester/transesterification reactions was studied.^177^ With a 92.30% oil transformation during the transesterification process of soybean oil and a full FFA conversion during the esterification of FAA at 120 °C, the AIL/HPMo/MIL-100(Fe) catalyst flashed outstanding performance. Additionally, the catalyst exhibited exceptional structural properties, strong acidic activity, and superior catalytic efficiency. Its recyclability was noteworthy, maintaining high oil conversion rates even after five cycles without significant degradation in performance. Li *et al.* studied Co-doped MIL-100(Fe) as a model catalyst incorporating (NH_4_)_2_SO_4_ selected for its role as a safe sulfiding agent in acid catalysis.^185^ When used in the synthesis of biodiesel, the resultant catalyst, known as MS(Fe/Co), achieved a 92.56% conversion rate for OA at 70 °C in two hours. In order to create Ni/MIL-96(Al), Aisyah *et al.* investigated the addition of different nickel loadings to MIL-96(Al) using the beginning wetness impregnation method.^186^ The catalyst's Lewis acid properties were strengthened by the addition of Ni, which greatly increased the catalyst's catalytic activity. The most efficient catalyst among those tested was 3% Ni/MIL-96(Al), which produced biodiesel by transesterifying crude palm oil and methanol at a rate of 85.24%.

**Fig. 8:**
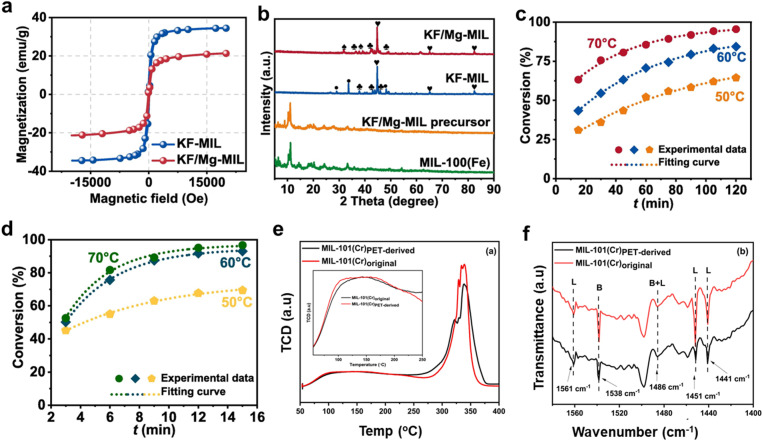
(a) Magnetization profiles of KF-MIL and KF/Mg-MIL. (b) XRD spectra of MIL-100(Fe), KF/Mg-MIL, KF-MIL, KF/Mg-MIL samples. (c and d) Conversion rate of KF/Mg-MIL in WB and MW at different reaction temperatures. This figure has been adapted/reproduced from ref. 187 with permission from Elsevier Ltd, Copyright 2024. (e) NH_3_-TPD and (f) and pyridine-FTIR spectra for MIL-101(Cr) samples. This figure has been adapted/reproduced from ref. 176 with permission from Elsevier Ltd, Copyright 2024.

A group of alkaline catalysts, particularly KF/Mg-MIL, with potent microwave absorption properties, was synthesized, and their impact on transesterification for the synthesis of biodiesel was examined.^187^ The study found that the microwave absorbing capability, rather than the previously recognized basicity, played a dominant role in promoting the reaction, with KF/Mg-MIL exhibiting high microwave absorption properties. When equated to conventional water bath heating (WB), these catalysts effectively transformed microwave energy into thermal power through loss of dielectric and magnetic loss, saving 50% of the energy and reducing CO_2_ emissions by 1051.61 kg per ton of biodiesel. The catalytic performance in the production of biodiesel was further enhanced by a “non-thermal” effect with KF/Mg-MIL under microwave heating, which decreased the *E*_a_ by 2.49 kJ mol^−1^ and raised the frequency factor by 793.32 min^−1^. The magnetic properties of KF-MIL and KF/Mg-MIL are paramagnetic with very little coercivity and remanence (Fig. 8a). In comparison to KF/Mg-MIL (21.32 emu g^−1^), KF-MIL has a higher saturated magnetization (33.44 emu g^−1^), which improves its capacity to store and release magnetic energy. This enables effective catalyst isolation using an external magnetic field. From Fig. 8b, the XRD spectrum of the KF/Mg-MIL precursor closely resembles that of MIL-100(Fe), suggesting the retention of the phase structure once loading KF and Mg(OAc)_2_. Furthermore, no distinct peaks for KF and Mg(OAc)_2_ were observed in the precursor, indicating their homogeneous distribution within the MIL-100(Fe) structure. However, during the activation process, the original MIL-100(Fe) structure was altered, and the organic linker was carbonized, revealing KF at 29.03° and 33.63° in the final KF-MIL. Additionally, KMgF_3_ appeared at 31.77° and 39.14° only in the KF/Mg-MIL sample, confirming its formation. The SEM and TEM analyses of KF-MIL and KF/Mg-MIL are studied, respectively. The SEM images show that KF-MIL has a flake-like shape, enhancing microwave scattering through piled-up pores. TEM images confirm the uniform distribution of Fe particles. In contrast, the SEM images of KF/Mg-MIL have a rough, irregular morphology with more compact particles, leading to a lower surface area. This results in more heterojunction interfaces between metal and carbon, explaining the higher *ε′* and *ε″* values of KF/Mg-MIL. Fig. 8c and d presents the conversion of KF/Mg-MIL at varying temperatures. The water bath (WB) method (Fig. 8c) achieved a maximum conversion of 95.48% at 70 °C. In comparison, the microwave (MW) method (Fig. 8d) reached a higher conversion of 99.20% at a lower temperature of 65 °C, demonstrating that MW is more efficient in achieving higher conversion at reduced temperatures compared to WB.

In this context, MIL-101(Cr) was made from PET bottles and used for biodiesel synthesis, a renewable energy alternative to fossil fuels, by esterifying OA with methanol.^176^ At a 1 : 39 molar ratio of OA to methanol, 6 wt% catalyst amount, 65 °C, and a 4-hour reaction time, the greatest biodiesel yields (86.9% and 80% for MIL-101(Cr) from pristine and dump PET bottles, individually) were obtained. According to the kinetic analysis, the MIL-101(Cr)PET-derived had activation energies of 28.3 kJ mol^−1^, and the original MIL-101(Cr) had 25.27 kJ mol^−1^. Furthermore, the catalyst's potential for sustainable biodiesel production was demonstrated by the fact that the waste-derived MIL-101(Cr) was successfully reused for three cycles. In contrast, the original MIL-101(Cr) catalyst was effective for up to five cycles. By comparing the acidity of the MIL-101(Cr) original and MIL-101(Cr)PET-derived families using NH_3_-TPD, as shown in Fig. 8e, the reaction mechanism's driving force was revealed. MIL-101(Cr)PET-derived has medium acid sites (150–200 °C) from Cr–OH_2_ and Cr–OH centres and strong acid sites (>325 °C) from unsaturated Cr metal sites, which are advantageous for catalysis, especially in the production of methyl oleate (Fig. 8e). Lewis acid sites are also confirmed to be present in both materials by Fig. 8f, which shows distinctive absorption bands at 1451 cm^−1^ and 1561 cm^−1^ that are suggestive of unsaturated Cr species. In conclusion, the unsaturated Cr(iii) Lewis acid sites play a crucial role in the catalytic activity of both MIL-101(Cr) original and MIL-101(Cr)PET-derived materials to produce methyl oleate from OA. Table 3 provides an overview of recent advances in utilizing acid and alkaline MIL-MOFs for biodiesel synthesis.

**Table 3:** Summarization of recent progress of using MIL-MOFs for biodiesel production

MIL-MOFs catalyst	Feedstock	Alcohol : oil	Catalyst loading	Temp (°C)	Time (h)	Reactor mode	Biodiesel yield (%)	Cycles	Acid/Base density	Ref.
MIL-100(Fe)–SO_3_H	OA	10 : 1	8 wt%	100 (reflux)	4	Batch (flask)	95.86	5	(Lewis Fe + SO_3_H)	182
[SO_3_H–CH_2_)_3_-HIM]_3_PW_12_O_40_-MIL-100	OA	15 : 1	5 wt%	90	3 (ultrasound + stir)	Batch (ultrasound + stir)	94.60	6	1.74 mmol g^−1^ acidity	123
AIL/HPMo/MIL-100(Fe)	Acidic soybean oil	30 : 1 Me	5 wt%	120	3 (sealed)	Batch (one-pot)	92.30	5	Enhanced Brønsted + Lewis	177
SIL-PW/MIL-101(Cr)	OA	8 : 1	10 wt%	70	4 (optimized)	Batch (flask)	94.30	5	(Brønsted + Lewis)	184
Fe@C–Sr	Palm	9 : 1	3 wt%	65	2 (stirred)	Batch (reflux)	91.93	6	(Basic SrO sites)	178
3 %Ni/MIL-96(Al)	Crude palm oil	12 : 1	3 wt%	180	1 (autoclave)	Batch (autoclave)	85.24	3	—	186
MIL-100(Fe) and calcium acetate	Palm	12 : 1	5 wt%	65	1	Batch (reflux)	97.00	5	(Basic CaO sites)	272
CoFe_2_O_4_/MIL-88B(Fe)–NH_2_/(Py-Ps) PMo	Soybean oil	20 : 1	5 wt%	95	2 (microwave)	Microwave batch	95.60	5	(Brønsted + Lewis)	181
(AIL/HPMo/MIL-100(Fe))	Soybean oil	30 : 1	9 wt%	120	6 (92.3% oil conversion; FFA ∼100%); also reported at 8 (92.8%)	Batch	92.30	5	2.68 meq H^+^/g (Brønsted + Lewis	177
NH_2-_MIL-101- Sal-Zr	OA	6 : 1	8 wt%	110	8	Batch (reflux)	74.10	3	—	273
SrO-MIL-100 (Fe)	Palm	6 : 1	2 wt%	65	0.5	Batch (reflux)	96.19	????	(Basic SrO, leaching observed)	179
CaO/MIL–100(Fe)	Palm oil	6 : 1	2 wt%	65	1	Batch (reflux)	95.07	4	(Basic CaO)	180

### 6.3 ZIFs-MOF for biodiesel production

The use of MOFs with zeolite-like structures, referred to as zeolitic imidazolate frameworks (ZIFs), in the production of biodiesel has been the subject of recent studies. The remarkable hydrothermal, chemical, and thermal stability of ZIFs is well known.^188^ Usually, imidazole or its derivatives in solutions with multivalent ions are used to create these frameworks.^189–193^ One noteworthy example is ZIF-8 with strong structural and chemical stability, a large surface area, and adjustable pore sizes. These characteristics make ZIF-8 popular in applications like biodiesel production (Table 4).

**Table 4:** A summary of the latest developments in the use of ZIF-MOFs catalysts for the creation of biodiesel

ZIF-MOF catalysts	Feedstock (FFA%, water %)	Alcohol : oil	Catalyst loading	Temp (°C)	Time (h)	Reactor mode	Biodiesel yield (%)	Cycles	Acid/Base density	Ref.
CaO/ZnO ZIF-8 MOF	Soybean oil	12 : 1	5 wt%	65	0.75	Batch (microwave)	97.40	5	(Basic CaO sites)	195
Lipase@ZIF-8	Hemp oil	—	20 mg	40	24	Batch (shaking)	75.00	4	(Biocatalyst)	198
(ZIF-8) impregnated with NaOH	Vegetable oil	6 : 1	3 wt%	65	1.5	Batch (reflux)	70.00	4	(Basic OH−)	199
SnO_2_ @Mn-ZIF	Seed oil	18 : 1	5 wt%	150	4	Batch (autoclave)	91.50	5	(Acid WO_3_, base SnO_2_)	200
{Mo_132_} supported on SOM-ZIF-8	20% OA + soybean oil	20 : 1	5 wt%	120	8	Batch (sealed)	93.90	5	(Brønsted POM sites)	197
BPB/Fe_3_O_4_/ZIF-67@K_2_CO_3_	WCOT	12 : 1	9 wt%	65	3	Batch (reflux)	99.18	5	(Strong base K_2_CO_3_)	212
HPW@ZIF-67	Microalgal lipids	15 : 1	10 wt%	90	4	Batch (reflux)	98.50	4	1.25 mmol H^+^ g^−1^ (–SO_3_H density)	214
C–Zn/Co-ZIF = 4	Microalgal lipids	9 : 1	8 wt%	70	2	Batch (reflux)	96.20	5	(N–C sites, Co metal)	201
SnPW@ZIF-8	Soybean oil	12 : 1	10 wt%	65	4	Batch (reflux)	92.80	5	(Brønsted–Lewis acids)	194
NaOH/magnetized ZIF-8	Vegetable	6 : 1	5 wt%	60	2	Batch (reflux)	1.00	—	(Insufficient base sites)	217
KNa/ZIF-8	Soybean	6 : 1	5 wt%	60	4	Batch (reflux)	3.50	—	(Weak base doping)	193
KNa/ZIF-8@GO	Soybean	6 : 1	5 wt%	65	4	Batch (reflux)	3.00	—	(No improvement *vs.* above)	203
Co-MOF	Erythrina Mexicana	9 : 1	10 wt%	120	3	Batch (autoclave)	83.70	3	(Lewis acid Co sites)	248

**Fig. 9:**
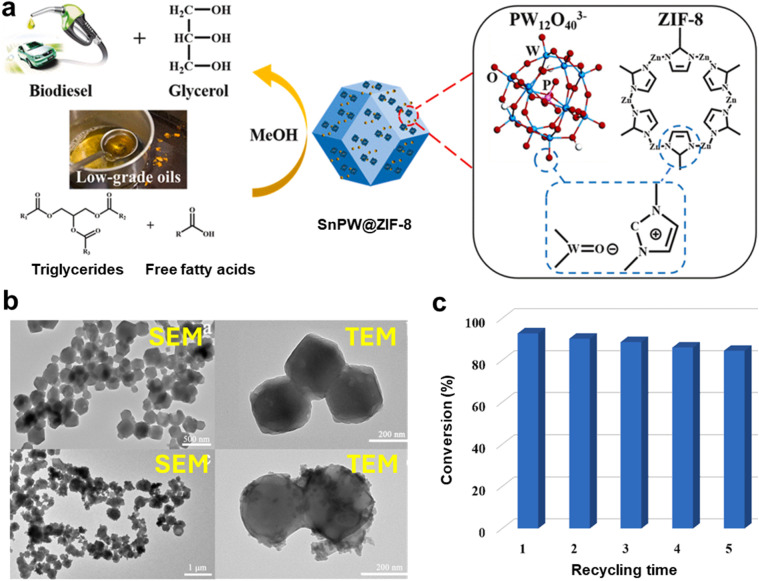
(a) A representation of the SnPW@ZIF-8 catalyst's synthesis. (b) SEM and TEM images of ZIF-8 and 0.5SnPW@ZIF-8. (c) Reusing the catalyst 0.5SnOW@ZIF-8 in oil transesterification. This figure has been adapted/reproduced from ref. 194 with permission from Elsevier Ltd, Copyright 2024.

A catalyst called SnPW@ZIF-8 was created to produce biodiesel from inferior acidic oils.^194^ This catalyst improves the processes of oil transesterification and FAA esterification by combining tin metal (Sn) with ZIF-8. Under ideal conditions (140 °C for 10 hours, 35 : 1 MTOR, and 6 wt% catalyst loading), SnPW@ZIF-8 demonstrated an impressive 92.80% oil conversion and 97.30% FAA conversion thanks to its two Brønsted–Lewis acid sites and large surface area. Furthermore, the catalyst showed promise for producing sustainable biodiesel from acidic oils by maintaining exceptional catalytic efficiency throughout the five runs. Fig. 9 presents the synthesis and characterization of the SnPW@ZIF-8 catalyst. Fig. 9a illustrates the schematic synthesis process. The SEM and TEM analyses collectively revealed that the 0.5SnPW@ZIF-8 catalyst exhibited a noticeably roughened surface morphology, with TEM showing a compact dark region corresponding to ZIF-8 and a loose bright region attributed to SnPW, confirming the successful deposition of SnPW on the MOF surface (Fig. 9b). Furthermore, the corrugated pattern of the incorporated SnPW observed *via* TEM, along with the uniform nanoparticle structure identified by SEM, highlighted the exposure of abundant active sites, which are crucial for enhancing the catalytic efficiency in oil transesterification reactions. Fig. 9c demonstrates the catalyst's excellent performance and stability as it maintains its efficiency over five consecutive reaction cycles. The strong interactions between the SnPW species and the imidazole groups of ZIF-8 effectively prevent leaching of the active SnPW, significantly enhancing the catalyst's stability and reusability.

**Fig. 10:**
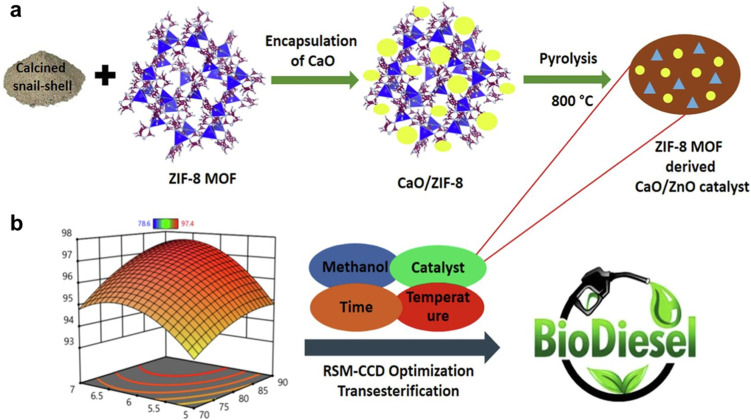
(a) Preparation steps for the ZIF-8 MOF-derived CaO/ZnO catalyst. (b) 3D surface curve with relation to catalyst wt, time, MTOR, and temperature. This figure has been adapted/reproduced from ref. 195 with permission from Elsevier Ltd, Copyright 2024.

L. Ruatpuia *et al.* employed ZIF-8 MOF and snail shell-derived biomass to create a composite CaO/ZnO nanostructure catalyst.^195^ Thermodynamic studies confirmed the endothermic nature of the transesterification process. The optimized transesterification conditions at 90 °C for 50 minutes with a 1 : 20 molar ratio of soybean oil to methanol and a 7 wt% catalyst load yielded 97.40% biodiesel. The catalyst also demonstrated reusability after five cycles. Additionally, the study included a life cycle cost analysis (LCCA) to evaluate the economic feasibility of scaling biodiesel production. The schematic of the preparation steps for the ZIF-8 MOF-derived CaO/ZnO catalyst is depicted in Fig. 10a. The 3D surface plots in Fig. 10b demonstrate significant interactions between key parameters such as catalyst wt, reaction time, MTOR, and temperature, with optimal FAME yields achieved at specific combinations (*e.g.*, 20 : 1 MTOR, 7 wt% catalyst, 90 °C, and 50 min). The results highlight the importance of balancing these factors to maximize biodiesel production while avoiding declines caused by prolonged reaction times or excessive temperatures. In further advancements, a new MOF, LeZIF-8-PAX, was developed by incorporating phthalic acid as an exchange ligand. This modification enhanced the structural stability and catalytic activity of ZIF-8. LeZIF-8-PAX showed dual stability in both pure OA and aqueous environments, along with increased specific catalytic activity. FTIR analysis revealed improved structural flexibility compared to ZIF-8. The modified MOF, ET 2.0/LeZIF-8-PA0.5, retained its original morphology and catalytic performance after five batch reactions in biodiesel production.^196^

**Fig. 11:**
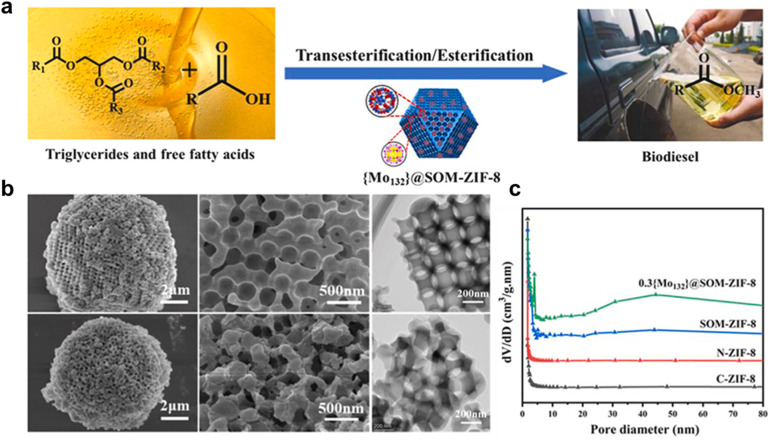
(a) Schematic for the transesterification reaction over {Mo132}@SOM-ZIF-8. (b) SEM and TEM images for{Mo132}@SOM-ZIF-8. (c) Pore size distributions of samples. This figure has been adapted/reproduced from ref. 197 with permission from Elsevier Ltd, Copyright 2024.

To synthesize biodiesel from acidic oils, a new catalyst with hierarchical porous structures was created. With a high surface area and two Brönsted-Lewis acid sites, it converted 93.90% of the oil at 130 °C in 8 hours with a 2 wt% catalyst dosage.^197^ The schematic representation for the {Mo132}@SOM-ZIF-8 catalyst for the biodiesel synthesis is shown in Fig. 11a. The key findings from the specified SEM and TEM images (Fig. 11b) reveal that the SOM-ZIF-8 support exhibits a highly ordered honeycomb-like macroporous structure with spherical particles of 2–10 µm and smooth surfaces, facilitating efficient mass transfer for large molecules. After {Mo132} doping, the hierarchical porous architecture remains intact, confirming structural stability. TEM analysis further validates the ordered macropores (≈160 nm) inherited from the PS template, with interconnected channels enhancing substrate accessibility. Elemental mapping confirms uniform distribution of O, C, N, Zn, and Mo, verifying successful integration of {Mo132} clusters. These features collectively minimize diffusion barriers and expose active sites, significantly improving catalytic performance in transesterification reactions. Fig. 11c shows that the 0.3Mo132SOM-ZIF-8 catalyst retains the microporous structure of SOM-ZIF-8 while introducing mesopores upon Mo132 loading, enhancing structural complexity and catalytic efficiency.

**Fig. 12:**
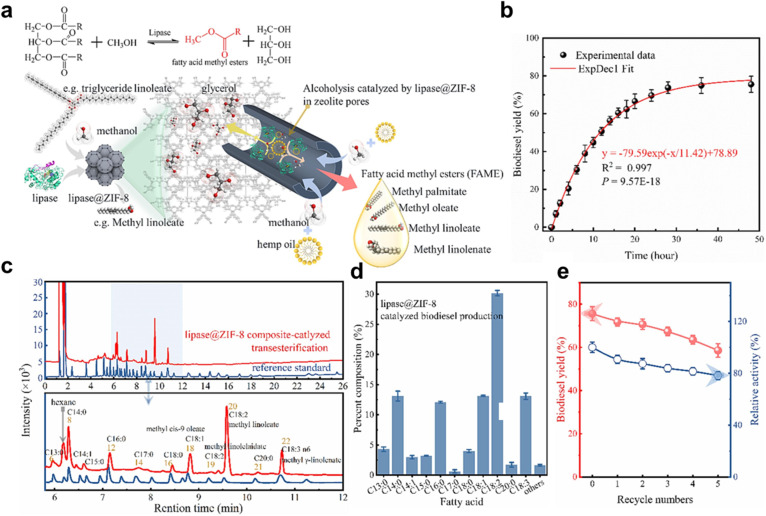
(a) Graphical representation of biodiesel production *via* lipase@ZIF-8. (b) Influence of reaction duration and moisture content on biodiesel production yield. (c) FAME composition is derived from hemp oil. (d) Fatty acid distribution in FAME products generated through lipase-driven transesterification. (e) Recyclability assessment of lipase@ZIF-8 biocomposites catalysts. This figure has been adapted/reproduced from ref. 198 with permission from Elsevier Ltd, Copyright 2024.

Due to their protective coating, these biocomposites have better pH and thermal stability. Highly porous lipase-loaded ZIF-8 biocomposites were created by F. Zha *et al.* using an *in situ* self-assembly technique.^198^ The flexibility of lipase architectures is increased by these biocomposites’ decreased activation energy and improved substrate sensitivity. It took about 36 hours to reach a maximum yield of 75% under ideal conditions (50 °C, MTOR 6 : 1). For the synthesis of biodiesel, Lipase@ZIF-8 biocatalysts showed outstanding operational stability and efficient reusability. Fig. 12a illustrates the lipase-catalyzed transesterification of hemp oil with methanol for biodiesel synthesis. The logistic model effectively described biodiesel yield kinetics, achieving a maximum of ∼75% at 36 h under optimized conditions (50 °C, 1 : 6 oil/methanol ratio) (Fig. 12b). Though further optimization of parameters like water content, methanol feeding strategy, and enzyme loading are critical to enhance yield and process efficiency. GC analysis revealed the fatty acid composition of hemp oil, dominated by linoleic (47.57%) and linolenic acids (16.48%), enabling calculation of its average molecular weight (870 g mol^−1^) (Fig. 12c). This is further detailed in Fig. 12d, where the lipase@ZIF-8-catalyzed biodiesel retained a FA profile similar to hemp oil, with methyl linoleate (30.19%) as the major product, but showed lower conversion of linoleic acid, indicating substrate selectivity toward shorter-chain saturated fatty acids. Finally, Lipase@ZIF-8 biocomposites demonstrated high reusability, retaining 80% initial activity and 60% conversion efficiency after five cycles, attributed to ZIF-8's protective matrix stabilizing the enzyme, making it a promising biocatalyst for biodiesel production (Fig. 12e).

Another study produced biodiesel meeting American Society for Testing and Material (ASTM) standards with a cetane number 15% higher than the ASTM maximum.^199^ This was achieved using a catalyst comprising a NaOH-impregnated magnetized ZIF-8 framework, which delivered 70% conversion efficiency in the ethanolysis of vegetable oils. A reaction temperature of 75 °C, a catalyst loading of 1%, a reaction duration of 90 minutes, and an ethanol/oil moles proportion of 21 : 1 were all considered optimal.

A bifunctional heterogeneous catalyst in biodiesel production simultaneously facilitates esterification (converting FAA) and transesterification (converting TGAs), enhancing reaction efficiency, simplifying purification, and enabling catalyst reuse due to its solid-phase nature. In this context, Juma *et al.* developed a bifunctional heterogeneous catalyst with tungstophosphoric acid (TPA) moieties and SnO_2_@Mn-ZIF core–shell support.^200^ The catalyst produced an excellent biodiesel yield of 91.50% under ideal conditions (methanol/oil molar proportion 18 : 1, catalyst load 6%, stirring speed 500 rpm, reaction temperature 100 °C for 3 hours).

Also, to convert microorganism lipids into biodiesel, researchers developed a bifunctional acid-based catalyst, C–Zn/Co-ZIF = 4, enhancing catalytic performance *via* pyrolysis. Zn doping minimized catalyst size, and DFT calculations demonstrated the superior performance of non-saturated metal N_*x*_. This catalyst achieved a 96.20% efficiency for microalgal biodiesel production.^201^ Researchers also synthesized Ce–Cr/ZIF-8, a reusable bimetal-doped catalyst, achieving a biodiesel yield of 92.6% with optimized doping concentrations and reaction parameters. By decreasing viscosity and diffusion resistance, raising the reaction temperature from 50 to 65 °C increased the yield of biodiesel.^202^ KNa/ZIF-8 nanocomposites, synthesized hydrothermally, catalyzed soybean oil transesterification with remarkable efficiency. Using optimal conditions (MTOR 10 : 1, temperature 65 °C, catalyst load 8%, reaction duration 3.5 hours), a 98% conversion rate was achieved.^203^

**Fig. 13:**
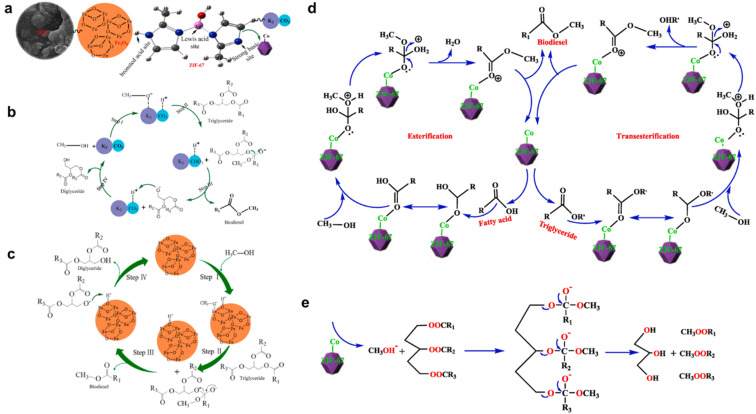
(a) Schematic illustration of the BPB/Fe_3_O_4_/ZIF-67@K_2_CO_3_ nanocatalyst structure. The roles of its components in biodiesel synthesis: (b) K_2_CO_3_, (c) Fe_3_O_4_, (d) ZIF-67’s acidic sites, (e) ZIF-67’s. This figure has been adapted/reproduced from ref. 212 with permission from Elsevier Ltd, Copyright 2024.

Waste recycling is important because it conserves natural resources, reduces pollution and greenhouse gas emissions, saves energy, minimizes landfill waste, and supports a healthier environment and sustainable economy.^47,204–206^ Recent research highlights the burgeoning potential of biochar and waste-derived carbon materials in diverse applications, including environmental remediation, energy storage, and catalysis.^142,206–211^ Biochar banana peels (BPB)/Fe_3_O_4_/ZIF-67@K_2_CO_3_ showed optimal production at 65 °C for biodiesel synthesis from waste cooking oil, with reaction times of 3 and 4 hours for treated and untreated oil, respectively. SEM, XRD, and TEM were used to characterize the catalyst and confirm its effectiveness.^212^ The authors are deeply interested in elucidating the mechanistic intricacies of the transesterification process for biodiesel synthesis, particularly leveraging the multifunctional architecture of the BPB/Fe_3_O_4_/ZIF-67@K_2_CO_3_ nanocatalyst (Fig. 13a). This study underscores the synergistic interplay of the catalyst's components, each contributing distinctively to the reaction pathway. The mesoporous structure, confirmed *via* BET analysis, enhances accessibility to active sites, while K_2_CO_3_ on the catalyst's surface generates nucleophilic methoxide ions (CH_3_O^−^) that initiate triglyceride cleavage (Fig. 13b). The Fe_3_O_4_ magnetic nanoparticles provide dual Lewis acid (metal ions) and Brønsted base (oxygen sites) functionalities, facilitating methanol activation and biodiesel formation (Fig. 13c). ZIF-67, with its tunable acidic and basic sites, drives both trans/esterification reactions by stabilizing carbo-cations and enabling tetrahedral intermediate formation (Fig. 13d), while its basic sites mirror the role of K_2_CO_3_ in methoxide-mediated pathways (Fig. 13e). Additionally, BPB's functional groups (C–O, C=O) further promote methoxide generation, reinforcing the catalyst's efficiency. The integration of these components not only accelerates reaction kinetics but also ensures catalyst regeneration, highlighting the nanocatalyst's robustness for sustainable biodiesel synthesis. This mechanistic understanding bridges the gap between catalyst design and industrial scalability, offering a blueprint for optimizing multifunctional catalysts in renewable energy applications.

**Fig. 14:**
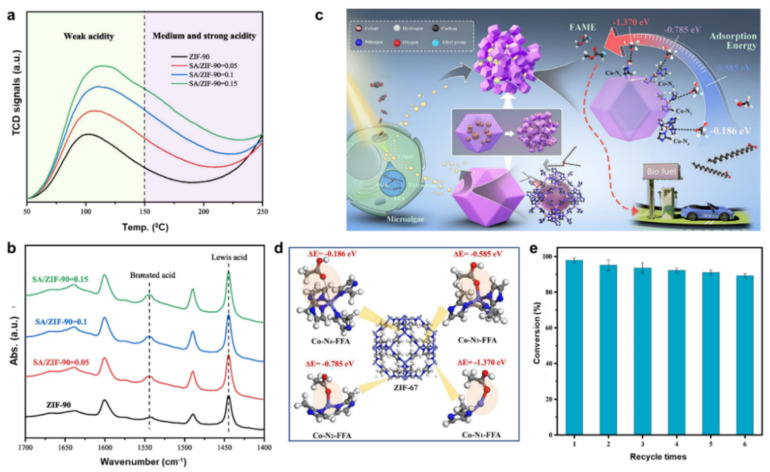
(a) NH_3_-TPD curves of ZIF-90 and SA/ZIF-90 catalysts. (b) ZIF-90 and SA/ZIF-90 pyridine bands. This figure has been adapted/reproduced from ref. 213 with permission from Elsevier Ltd, Copyright 2023. (c) Production of biodiesel from microalgal lipids using ZIF-90 catalysts. (d) DFT and absorption energies of acetic acid on Co. (e) Recycling process of ZIF-90 catalysts. This figure has been adapted/reproduced from ref. 215 with permission from Elsevier Ltd, Copyright 2023.

In bifunctional catalysts, the Lewis acid sites efficiently catalyzed the transesterification of TGAs in microalgal lipids, while the Brønsted acid sites were more active for esterification reactions of FAA. The ZIF-90 was modified with sulfamic acid (SA) to create an effective bifunctional catalyst with synergistic Lewis and Brønsted acid centres. At 200 °C, biodiesel production from microalgae lipids increased from 80.6% to 98.3% when the ZIF-90 weight ratio to SA was 0.05, making it an ideal catalyst. Even after six reusability cycles, the conversion efficiency remained high at 91.7%.^213^ This study elucidated the critical role of catalyst acidity in enhancing transesterification and esterification efficiency, as demonstrated through Py-IR and NH_3_-TPD analyses (Fig. 14a and b). The findings revealed that ZIF-90 primarily exhibited weak acid sites (<150 °C), attributed to unsaturated Zn^2+^ coordination. However, incorporating SA into ZIF-90 significantly amplified both total acidity and the proportion of medium-strong acid sites (>150 °C), reaching 1.457 mmol g^−1^ for SA/ZIF-90 (0.15 loading) compared to 0.478 mmol g^−1^ for pristine ZIF-90. This enhancement stemmed from the introduction of SA groups and the disruption of Zn–N bonds, which generated additional Lewis acid sites *via* exposed Zn^2+^ and imidazole *N*-terminal. Notably, the ratio of medium-strong to weak acidity surged from 5.75% to 34.16% with SA loading, underscoring the dual contribution of Brønsted and Lewis acid sites to catalytic activity. These results highlight SA/ZIF-90 as a promising catalyst for acid–sensitive reactions, where tunable acidity directly correlates with performance efficiency. The HPW/ZIF-67 catalyst achieved a maximum catalytic conversion efficiency of 98.5% for converting microalgal lipids into biodiesel.^214^ Compared to pure ZIF-67, which had a conversion efficiency of only 72.8%, the HPW/ZIF-67 catalyst demonstrated significantly higher activity due to its enhanced Brønsted acid and Lewis acid-base properties.

Another study aimed to synthesize an ultrafine ZIF-67 with unsaturated cobalt-nitrogen active sites (Co-N_*x*_) to catalyze the efficient conversion of high acid-value microalgal lipids into biodiesel.^215^ The graphical abstract illustrates the synthesis of an ultrafine ZIF-67 with unsaturated Co-N_*x*_ for the catalytic conversion of microalgal lipids to biodiesel (Fig. 14c). The optimal catalyst demonstrated conversion efficiencies of 99.3% for FAA and 97.7% for TGAs when converting acidic lipid feedstocks into biodiesel. DFT calculations, illustrated in Fig. 14d, elucidated the adsorption behavior of acetic acid, a model compound for FAA on Co-N_*x*_ within the ZIF-67 catalyst. The adsorption energies varied significantly across different coordination environments: the Co–N_4_-site exhibited the weakest interaction (−0.186 eV), attributed to steric hindrance limiting effective adsorbate binding. In contrast, Co–N_3_, Co–N_2_, and Co–N_1_ demonstrated progressively stronger adsorption, with energies of −0.585, −0.785, and −1.370 eV, respectively. Notably, the strongest interaction occurred at the Co–N_1_-site, where enhanced electron transfer from the catalyst to acetic acid increased the electropositivity of the carbonyl carbon from 1.379 eV to 1.537 eV. This electronic modulation significantly facilitated nucleophilic attack by methanol, thereby accelerating the esterification reaction critical for biodiesel synthesis. The systematic analysis underscores the pivotal role of coordinatively unsaturated Co-N_*x*_ sites in optimizing catalytic activity through electronic and steric effects. Fig. 14e demonstrates the excellent recyclability of the ZIF-67 catalyst, maintaining a catalytic conversion efficiency of 89.3% after 6 cycles. The slight reduction in efficiency over time is attributed to the occupation of the catalyst's active sites by impurities from microalgal lipids, which reduces its catalytic activity.

**Fig. 15:**
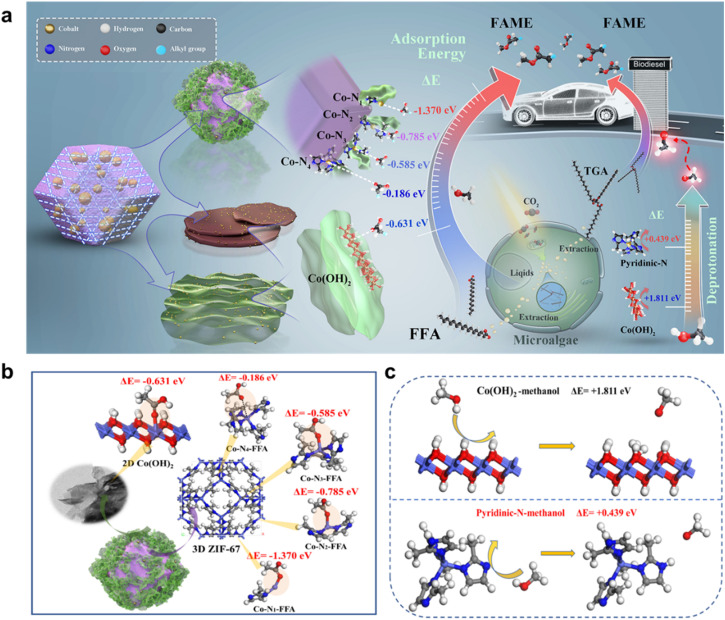
(a) Structural optimization of ZIF-67 highlighting active metal sites for lipid-to-biodiesel conversion. (b) Adsorption energies of acetic acid (FFA model) on Lewis acidic Co sites. (c) Free energy profile of methanol deprotonation on basic sites. This figure has been adapted/reproduced from ref. 216 with permission from Elsevier Ltd, Copyright 2022.

According to previous research by H. Guo *et al.*, a core–shell structure of ZIF-67 with 2D ultrathin cobalt-based nanosheets as the shell to expose more active metal sites and enhance the acid-base properties of the catalyst, thereby improving the catalytic conversion efficiency of acidic microalgal lipids to biodiesel (Fig. 15a).^216^ The structure offers several advantages: (i) an open framework exposes more active sites, (ii) increased surface area and structural defects enhance acid-base properties, and (iii) higher numbers of unsaturated metal and pyridinic-N sites. The optimal catalyst demonstrated an efficiency of 96.10% in catalyzing the conversion of microalgal lipids to biodiesel, significantly surpassing the performance of conventional ZIF-67 (75.4%). The DFT calculations revealed the catalytic activities of different cobalt coordination environments present in the catalysts. According to the results shown in Fig. 15b, the adsorption energies of acetic acid (a model for free fatty acids) on the coordinatively unsaturated CoN_1_, CoN_2_, and CoN_3_ sites were −1.370 eV, −0.785 eV, and −0.585 eV, respectively, indicating stronger interactions compared to the coordinatively saturated CoN_4_ site (−0.186 eV). This suggests that the unsaturated Co-N_*x*_ sites can more effectively activate the carbonyl carbon of the fatty acids, promoting the nucleophilic attack of methanol during the esterification reaction. Furthermore, the DFT calculations in Fig. 15c show that the free energy change for methanol deprotonation on the pyridinic-N sites (0.439 eV) is lower than that on the hydroxyl groups in Co(OH)_2_ (1.811 eV), indicating the pyridinic-N sites can more readily form methoxide anions to attack TGAs in the transesterification step.

The methanolysis technique employed a solid catalyst composed of NaOH and magnetized ZIF-8, which significantly improved the production of biodiesel. The FAME obtained met ASTM biodiesel standards. This method mirrored the ethanolysis process described earlier using the same catalyst,^199^ except ethanol was replaced with methanol. To promote fuel sustainability, the resultant biodiesel could be mixed with fossil diesel and used in diesel engines. With a catalyst-to-oil ratio of 3 wt%, an MTOR of 21 : 1, and a reaction time of only one hour at 65 °C, a nearly complete rate of conversion (∼100%) was attained.^217^

A CaO–ZrO_2_ heterogeneous catalyst made from MOFs was employed by Gouda *et al.* to transesterifying soybean oil, and following response-surface methodology (RSM) optimization, a maximum yield of 97.22% was obtained.^218^ When the catalyst's structural characteristics were examined using TEM, an even distribution of nanoscale particles with a typical particle size of 33.98 nm was revealed. In order to produce biodiesel, Giraldo *et al.* immobilized Candida Antarctica Lipase B (CALB) on ZIF-8 and MOF-199. Because of chemical interactions, CALB-MOF-199 was found to have better reusability, and the XRD spectra of the free MOF-199 and CALB-MOF-199 systems displayed distinct diffraction peaks that demonstrated the MOF's crystalline structure. The characteristic structure of MOF-199 was confirmed by the peaks matching those reported in the literature. The XRD spectrum changed slightly after Lipase was immobilized on ZIF-8 (CALB-ZIF-8), indicating that the immobilization of Lipase had little effect on ZIF-8's crystallinity.^219^

### 6.4 Ca-MOFs for biodiesel production

**Fig. 16:**
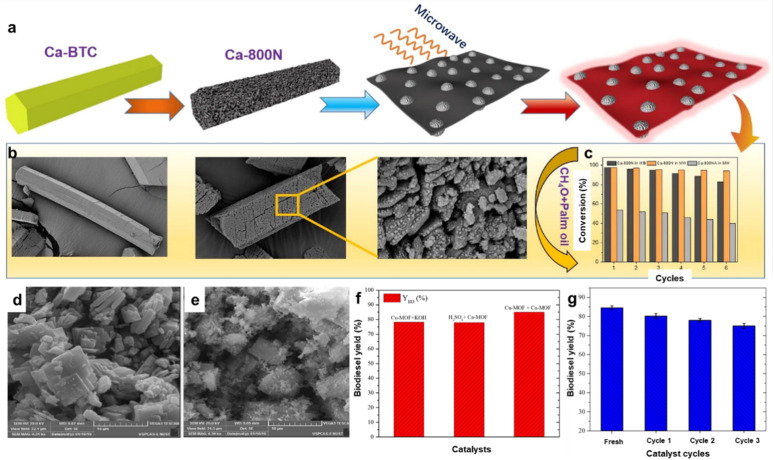
(a) The preparation and application of catalysts in the microwave field. (b) SEM images of Ca-BTC. (c) Comparison of the reusability of Ca–800 N, Ca-800NA in the MW field, and Ca–800 N in WB. This figure has been adapted/reproduced from ref. 223 with permission from Elsevier Ltd, Copyright 2022. SEM images of (d) Cu-MOF and (e) Ca-MO. (f) Comparison of catalyst performance in biodiesel yield. (g) Reusability of the catalyst over different cycles. This figure has been adapted/reproduced from ref. 106 with permission from Elsevier Ltd, Copyright 2022.

CaO is a powerful base catalyst that is most frequently used in the transesterification reaction to yield biodiesel from edible and non-edible oils.^220,221^ Nevertheless, CaO catalysts have limitations like low pore volume and surface area. To overcome these constraints, MOFs with calcium (Ca) as a metal site are created, providing stability, high surface area, and porosity, all of which are advantageous for the transesterification reaction.^222^ A recent study demonstrated that using microwave irradiation during the fabrication process of a nanosheet-like CaO/C catalyst derived from Ca-BTC led to increased catalytic activity in biodiesel production. XRD analysis confirmed the successful transformation of Ca-BTC into CaO at elevated temperatures, enhancing its catalytic performance. SEM-EDS analysis revealed the nanosheet structure of Ca–800 N with anchored CaO particles, which improved microwave absorption. TEM images further showed well-formed crystalline CaO particles embedded in carbon nanosheets, indicating a low graphitization degree.^223^Fig. 16a illustrates the catalyst preparation process, where Ca-BTC is produced and used as a precursor to create a highly stable microwave-targeted CaO catalyst, with CaO anchored in carbon nanosheets. Ca-BTC, with its hexagonal prism structure and smooth surface, is successfully synthesized, as shown in Fig. 16b. Fig. 16c shows that Ca–800 N maintains 94.14% conversion after 6 cycles, with only a 3.23% decrease in activity. In comparison, Ca-800NA's conversion drops from 53.91% to 40%, indicating the importance of carbon in Ca–800 N for microwave absorption.

MOFs based on copper and calcium were effectively created and used as catalysts to turn the residue of cooking oil into biodiesel. Cu-MOF and Ca-MOF showed crystal sizes of 49.1 nm and 20.8 nm, respectively, based on XRD analysis. Cu-MOF and Ca-MOF SEM images are shown in Fig. 16d and e, respectively. The Cu-MOF exhibited a highly crystalline cubic structure with an average size of ∼ 4–8 µm, while the Ca-MOF particles showed a cubic shape with an estimated size of 5–7 µm. Small unreacted particles were observed on the surfaces of both, which were later removed by washing.^106^ The study demonstrated that combining Cu-MOF and Ca-MOF catalysts achieved the highest biodiesel yield of 85% from waste cooking oil under optimized conditions (65 °C reaction temperature, 60-minute duration, and 1.0 g catalyst loading per step), as shown in Fig. 16f. This performance surpassed that of individual catalysts, with Cu-MOF yielding 78.3%. Ca-MOF yielding 78% under identical conditions. The enhanced efficiency of the dual catalyst system arises from a synergistic interplay between its acidic (Cu-MOF) and basic (Ca-MOF) properties: Cu-MOF facilitates the esterification of FAA. At the same time, Ca-MOF drives the transesterification of TGAs. This complementary activity addresses both key reaction pathways simultaneously, improving overall conversion efficiency compared to single-catalyst approaches. In the meantime, the first cycle shows an 80.2% biodiesel yield, with yields of 78% and 75.1% in the second and third cycles, respectively (Fig. 16g).

To address the limitations of CaO catalysts, such as Ca^2+^ leaching and poor pore structure in biodiesel production, a CaO/ZrO_2_ catalyst was synthesized by supporting calcium acetate on Zr-based UiO-66(Zr). Catalyst precursors were activated under nitrogen (UCN) and air (UCA) atmospheres, with calcination temperatures and calcium acetate loading optimized to enhance performance. Under transesterification conditions (65 °C, 60 min), the UCN650 catalyst (calcined at 650 °C in nitrogen) achieved a maximum oil conversion of 96.99% using 6 wt% catalysts and a 9 : 1 MTOR, retaining 92.76% conversion after three reuse cycles. In contrast, UCA700 (calcined at 700 °C in air) showed a lower initial conversion (92.94%) with 8 wt% catalyst loading. Characterization *via* XRD confirmed structural stability post-reaction, while XPS revealed UCN650's higher oxygen vacancy concentration and crystalline oxygen species, enhancing catalytic efficiency. This MOF-derived catalyst, with its simple synthesis and high reusability, demonstrates significant potential for sustainable biodiesel production, aligning with prior studies highlighting UiO-66(Zr)/CaO–ZrO_2_'s 96.99% yield under similar conditions.^134^

### 6.5 Cu-MOFs for biodiesel production

Cu-MOFs, or copper-based MOFs, have been thoroughly investigated as efficient catalysts for the esterification and transesterification processes that produce biodiesel.^156,224^ Cu-MOFs offer a sustainable, eco-friendly alternative to traditional catalysts, as summarized in the Table 5.^225^ During the transesterification of palm oil, Cu-BTC MOF produced a high FAME yield of 91%, indicating its potential as a useful catalyst to produce biodiesel. XRD analysis confirmed a highly crystalline structure with minimal amorphous regions, marked by a flat baseline and sharp peaks at specific 2*θ* values.^224^

**Table 5:** Summary of recent progress in using Ca-and Cu-MOF catalysts for biodiesel production

Ca-/Cu-MOFs catalyst	Feedstock	Alcohol : oil	Cat. Load	Temp (°C)	Time (h)	Biodiesel yield %	Cycles	Active sites	Ref.
CaO–ZrO_2_-MOF	Soybean	15 : 1	5 wt%	65	2	97.22	4	Basic O^2−^ sites CaO	218
CALB-MOF-199 & CALB-ZIF-8	African palm	3 : 1	40 mg (enzyme)	45	18	90.50	5	Biocatalyst	219
CuBTC	OA	20 : 1	10 wt%	160	6	78.60	3	Lewis Cu sites	68
Cu-MOF and Ca-MOF	WCO	18 : 1	8 wt%	75	3	85.00	5	Basic CaO, CuO	106
CaO/ZrO_2_ catalyst	Palm oil	12 : 1	5 wt%	65	2	92.94	5	CaO, ZrO_2_ basic	134
Ca-BTC	Palm oil	6 : 1	5 wt%	65	0.5	94.01	4	CaO nano-sheets	223
CuBTc	Palm oil	6 : 1	3 wt%	65	1	91.00	3	Lewis acid Cu	224
ILe@Cu@MOF	Xanthoceras sorbifolia	15 : 1	10 wt%	70	8	82.85	4	Brønsted IL, Cu sites	227
Copper(ii)-alginate beads	OA	6 : 1	5 wt%	80	4	71.80	5	Acidic alginate Cu	274
Cu_3_(BTC)_2_, FeCu-DMC	Furanic alcohols (furfural)	—	—	180	5	90.00	—	—	229
Cu/ZrO_2_-MOF	Glys (hydrogenolysis)	—	5 wt%	220	8	75.00	—	—	230
Cu-β-CD	Patr oil	20 : 1	15 wt%	65	4	88.63	4	Lewis Cu, base OH	275
Cu-BDC	Plastic waste	10 : 1	5 wt%	120	4	68.90	3	Lewis Cu	231
CuBTC	Palm oil	6 : 1	3 wt%	65	1	91.00	3	Lewis Cu	224
Fe_3_O_4_@HKUST-1	Soybean	6 : 1	5 wt%	65	2	92.30	5	Basic IL on Fe_3_O_4_	232

A study that used ILe@Cu@MOF to produce biodiesel *via* Xanthoceras sorbifolia Bunge oil revealed improved hydrophobicity and thermal stability. Functionalization with isoleucine (ILe) resulted in a decrease in the intensity of Cu-MOF's main diffraction peaks and shifts in diffraction angles, as evidenced by XRD, confirming successful functionalization. SEM images revealed flaky ILe structures attached to the Cu-MOF surface.^226^ The study demonstrates that the ILe@Cu@MOF catalyst realized a biodiesel yield of 82.85% under optimal conditions, showcasing its efficiency in the transesterification process. Zhang *et al.* synthesised an HPMo/Cu-BTC composite employing a one-pot hydrothermal process for producing biodiesel from OA and methanol *via* esterification with a high conversion of 93.7%.^227^ This composite exhibited excellent recyclability, maintaining over 80% catalytic activity after 7 cycles.

**Fig. 17:**
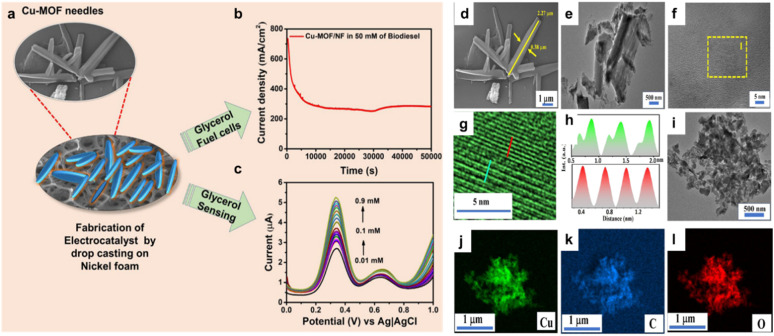
(a–c) Highlights the synthesis and application of Cu-MOFs as electrocatalysts in Gly sensing in biodiesel samples. (d and e) SEM and TEM images of Cu-MOF. (f–h) HRTEM of Cu-MOF catalyst. (i–l) EDS mapping of Cu-MOF and its elemental composition Cu, C, and O, respectively. This figure has been adapted/reproduced from ref. 228 with permission from American Chemical Society, Copyright 2024.

The Cu-MOF/NF electrocatalyst demonstrated a low limit of detection (LOD) of 0.172 µM, outstanding selectivity and sensitivity, and an outstanding current density of 690 mA cm^−2^. XRD analysis verified its well-defined crystalline structure, consistent with database entries.^228^ SEM images showed needle-like structures with a smooth surface, approximately 0.38 mm in diameter and 2–3 mm in length. The fabrication of the Cu-MOF-modified nickel foam electrode is shown in Fig. 17a. Fig. 17b shows that the electrocatalyst retained about 59% of its initial activity, indicating good stability. During chronoamperometry, the current density of biodiesel dropped significantly at the early stage due to the initial oxidation of biodiesel components, producing reactive intermediates. Fig. 17c shows that as the concentration of Gly increases, the current response of Cu-MOF/NF also increases, indicating that Gly initiates oxidation at a specific electrical potential. The functional groups in Gly readily adsorb onto the electrode surface, enhancing the DPV response of Cu-MOF/NF as more Gly molecules become available for oxidation. The SEM analysis revealed that the synthesized Cu-MOF comprises uniform needle-like structures with a columnar morphology, measuring approximately 0.38 µm in diameter and 2–3 µm in length (Fig. 17d). TEM imaging further confirmed the layered architecture and aligned needle ends, consistent with the SEM observations (Fig. 17e). HRTEM analysis (Fig. 17f–h) demonstrated crystalline order, with interatomic spacings of 0.32 nm (red line), 0.53 nm (cyan line), and 0.2 nm (green line), the latter attributed to moiré patterns from layered stacking. TEM micrographs (Fig. 17i) highlighted dispersed nanoscale needle structures. EDS mapping (Fig. 17j–l) confirmed the homogeneous distribution of Cu, C, and O, suggesting enhanced electrical conductivity due to uniform elemental integration. These findings collectively validate the structural integrity and compositional purity of the Cu-MOF.

Pd-supported Cu-MOFs, such as Pd/Cu-BTC, displayed high catalytic activity with over 90% yield for cyclopentanone compounds derived from 5-hydroxymethyl furfural and furfural. TEM analysis showed well-dispersed Pd nanoparticles in the pores of Cu-BTC. In contrast, Pd/FeCu-DMC exhibited larger Pd aggregates and lower catalytic performance due to weak Lewis acidity and a nonporous structure.^229^ Additionally, Cu/ZrO_2_ catalysts demonstrated promising results in converting Gly to 1,2-propanediol, contributing to greener manufacturing technologies. SEM analysis revealed compact and homogeneous morphologies for catalysts prepared *via* sol–gel zirconia, compared to granular structures in commercial zirconia-based catalysts.^230^

Cu-BDC MOFs synthesized from plastic waste were used for biodiesel production from coconut oil, achieving conversion rates of 43.75% for acids and 68.90% for biodiesel. XRD analysis confirmed a high degree of crystallinity, while SEM images revealed irregular cubic forms with unreacted particles on the surface.^231^ This study emphasizes Cu- and Ca-MOFs' intriguing potential for the synthesis of biodiesel, emphasizing the need for further optimization, performance enhancement, and industrial-scale applications. W. Xie *et al.* described the preparation of a magnetically recyclable solid base catalyst, Fe_3_O_4_@HKUST-1-ABILs, which was used for the transesterification of soybean oil with methanol to produce biodiesel, achieving a conversion of 92.3%.^232^

### 6.6 Enzymatic-modified MOFs for biodiesel production

**Fig. 18:**
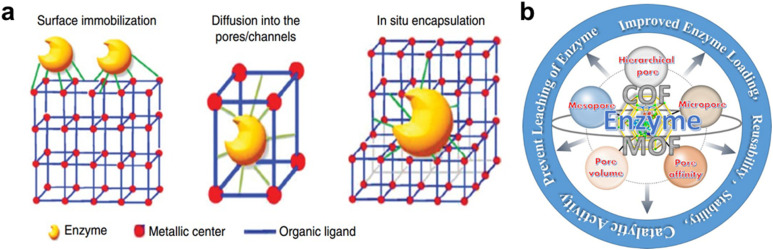
(a) Three key enzyme immobilization strategies within MOFs. (b) Schematic highlighting advantages of MOF-based enzyme immobilization. This figure has been adapted/reproduced from ref. 233 with permission from American Chemical Society, Copyright 2021.

Enzymatic modification of MOFs for biodiesel production represents a promising advancement in the field of renewable energy.^128^ MOFs, known for their high porosity and tunable structures, serve as excellent supports for enzyme immobilization, enhancing the efficiency and sustainability of biodiesel synthesis.^233,234^ The integration of enzymes into MOFs addresses the limitations of traditional catalysts by improving catalytic activity, stability, and reusability.^235,236^ J. Mehta *et al.* worked on enzyme immobilization using MOFs, highlighting their transformative potential as advanced platforms for biocatalyst design.^237^ By leveraging MOFs' exceptional surface area and tunable porosity, Mehta emphasizes their capacity for high enzyme loading while maintaining catalytic accessibility. Her review details three key immobilization strategies: surface incorporation, pore diffusion, and *in situ* encapsulation, each optimizing enzyme stability and reusability (Fig. 18a). C. Wang *et al.* review advancements in MOF- and COF-enzyme composites, emphasizing their tunable pore structures for enzyme encapsulation, protective roles in harsh environments, selective molecular diffusion, and critical enzyme–framework interactions while outlining future strategies to optimize these biomimetic systems with showing their advantages in the enzyme immobilization (Fig. 18b).^233^

This methodology not only optimizes the transesterification process but also aligns with the growing demand for green and sustainable energy solutions. The following sections delve into the specific aspects of enzymatic-modified MOFs for biodiesel production. Recent interest in polymers has surged due to their versatility in applications ranging from sustainable packaging and flexible electronics to biomedical implants and drug delivery systems.^142,148,149,238–241^ These materials can be engineered as robust supports in catalysis, enhancing stability and recyclability in biodiesel production while minimizing environmental impact.^205^ Zhong's study explored various nanomaterials for immobilizing enzymes and identified MOFs as superior candidates compared to silica nanoparticles, carbon nanotubes, and organic polymers.^242^ Enzyme-MOF catalysts have been the subject of extensive research because of their potential for producing biodiesel. To illustrate, within 12 h, B. Dalfard *et al.* produced about 83% biodiesel by immobilizing lipase within Zr-MOF/polyvinylpyrrolidone (PVP) nanofibrous composites.^243^

Y. Hu *et al.* immobilized *Aspergillus niger* lipase (ANL) onto a hydrophobically modified UiO-66 framework coated with polydimethylsiloxane (PDMS).^244^ Using soybean oil as feedstock, this system achieved a high biodiesel yield of 88%, attributed to PDMS-enhanced hydrophobic interactions that improved enzyme-MOF binding and catalytic efficiency. In a separate study, L. H. Liu *et al.* immobilized *Burkholderia cepacia* lipase (BCL) onto carbonized MIL-100(Al) (BCL@cMIL-100(Al)-800).^245^ The carbonized MOF that functionalized with carboxyl (–COOH) groups and optimized pore architecture facilitated superior enzyme loading and stability, yielding 80.3% biodiesel from soybean oil. Both works demonstrate how tailored MOF modifications, hydrophobic coatings, or carboxyl-functionalized carbon frameworks enhance enzyme immobilization and performance for sustainable biodiesel production.

In another study, biodiesel was synthesized from soybean oil in a nano-bioreactor without requiring a solvent, utilizing lipase@ZIF-67 and the fungus *Candida rugosa*, resulting in a 78% biodiesel yield. After six reaction cycles, the ZIF-67 carrier proved superior to silica gel and Celite-545.^246^ Additionally, encapsulating *Rhizomucor miehei lipase* (RML) within X-shaped ZIF-8 generated an enzymatic catalyst capable of achieving a biodiesel conversion rate of 95.6% due to its stable catalytic activity. Furthermore, the adsorption of ZIF-8 onto *Burkholderia cepacia* lipase (BCL) facilitated a biodiesel yield of 93.4%.^125^

**Fig. 19:**
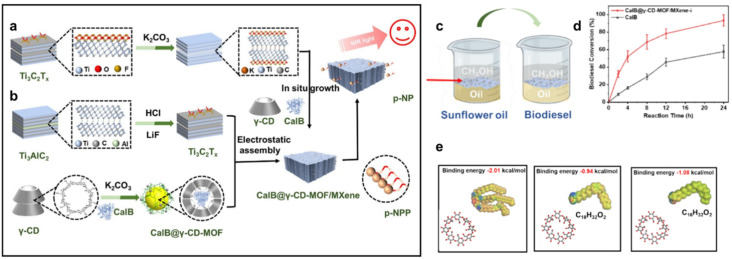
CalB@γ-CD-MOF/MXene synthesis (a) *In situ* growth method and (b) electrostatic assembly method. (c) Schematic diagram of biodiesel interfacial catalysis. (d) Biodiesel productivity of CalB@γ-CD-MOF/MXene-i over time. (e) DFT calculation of the cyclodextrins' binding energy to the substrate and R_1_C_18_H_32_O_2_, R_2_C_18_H_34_O_2,_ and R_3_C_16_H_32_O_2_. This figure has been adapted/reproduced from ref. 247 with permission from Elsevier Ltd, Copyright 2024.

An effective biodiesel catalysis method was created recently by encasing *Candida albicans lipase B* (CalB) in a γ-cyclodextrin MOF (γ-CD-MOF), which was stuffed with Ti_3_C_2_TX.^247^ Through interfacial enrichment and porous adsorption, this structure not only shielded the enzyme but also improved its catalytic activity. After six cycles, the CalB@γ-CD-MOF/MXene-i yielded 93.3% biodiesel conversion with 86.9% retained activity, demonstrating a 274.6% increase in activity under near-infrared (NIR) exposure. The synthesis pathway is illustrated in Fig. 19a and b. CalB@γ-CD-MOF/MXene composites were fabricated *via in situ* growth and electrostatic self-assembly. During *in situ* growth, CalB@γ-CD-MOF/MXene structures formed directly through the adsorption of precursor molecules onto K^+^-etched MXene surfaces, facilitated by electrostatic interactions. First, synthesizing CalB@γ-CD-MOF achieved electrostatic assembly and then performing electrostatic coprecipitation of the etched MXene. As shown in Fig. 19c, the biodiesel interfacial catalysis representation diagram for CalB@γ-CD-MOF/MXene-i is depicted. Fig. 19d illustrates the biodiesel productivity of CalB@γ-CD-MOF/MXene-i over time, with data presented as the mean value ± SD from three experiments. Fig. 19e shows the binding energy of cyclodextrins to the substrate (the primary ingredient in sunflower oil) and R_1_C_18_H_32_O_2_, R_2_C_18_H_34_O_2,_ and R_3_C_16_H_32_O_2,_ which were calculated using DFT. The table below summarizes the findings for enzymatic-modified MOF catalysts showcasing the diversity in catalyst performance (Table 6).

**Table 6:** Summarizing enzymatic modification of MOF catalysts for biodiesel production

Enzymatic modified MOF catalyst	Feedstock	Alcohol : oil	Enzyme loading	Temp (°C)	Time (h)	Reactor	Biodiesel yield %	Reusability	Ref.
ZIF-67@Lipase	Olive oil	3 : 1	20 mg (lipase PS)	40	24	Batch (stirred)	90.50	5	276
AOL@PDMS-ZIF-L	Soybean oil	4 : 1	15 mg (AOL lipase)	35	12	Batch (shake)	94.37	4	277
FA@ZIF-8-PCL, FA@MOF-199-PCL	African palm oil	3 : 1	30 mg (FA lipase)	40	18	Batch (rotary)	>90.00	5	278
2.0LeZIF-8-PA_0.5_	Soybean oil	4 : 1	2 wt% (lipase mix)	35	12	Batch (shake)	81.50	6	196
Zr-MOF/PVP@Lipase	Ricinus communis oil	4 : 1	20 mg (CALB lipase)	45	18	Batch (shake)	83.00	4	243
ROL@UiO-66-NH_2_	Waste frying oil & OA	5 : 1	25 mg (Rhizopus oryzae lipase)	40	24	Batch (shake)	82.05	5	279
CalB@γ-CD-MOF/MXene-i	Sunflower oil	3 : 1	30 mg (CALB enzyme)	35	10	Batch (LED + stir)	93.30	5	247

### 6.7 Other MOFs for biodiesel production

Heterogeneous catalysts like MOFs are widely preferred in biodiesel production due to their ease of separation compared to homogeneous alternatives.^100,108^ Among studied MOFs, including Hf-MOF, Mo-MOF, Ni-MOF, Co-MOF, and Zr-MOF, demonstrate their effectiveness in biodiesel production.^108,118^ P. Rodríguez *et al.* highlighted the acidic Co-MOF's performance, attributing its high catalytic activity to free carboxylic acid groups identified *via* XRD analysis.^248^ Co-MOF was synthesized through a hydrothermal reaction using Co(NO_3_)_2_·6H_2_O, 1,2-di-(4-pyridyl)-ethylene (L1), and 5-nitroisophthalic acid (L2) at 160 °C. This heterogeneous catalyst was applied to transesterifying Erythrina Mexicana oil for biodiesel production, with ultrasonication employed to enhance reaction efficiency. Under optimized conditions (60 °C, 12 hours), the catalyst achieved an 80% conversion rate, demonstrating its effectiveness. For the pre-esterification of *C. inophyllum* oil, Marso *et al.* investigated two kinds of MOFs: cobalt terephthalate (Co-Tp) and chromium terephthalate (Cr-Tp) as heterogeneous acid catalysts.^249^ Characterization methods such as FTIR, SEM, XRD, and thermogravimetry confirmed their catalytic performance, resulting in a 93% biodiesel yield under mild conditions without causing saponification. Al_2_O_3_ contributes to enhanced barrier, mechanical, and thermal properties of materials.^250^ S. Yu *et al.* evaluated Co-based catalysts supported on activated carbon (AC), Al_2_O_3_, and nitrogen-doped carbon (NC) for fatty acid hydrotreatment to produce green diesel.^251^ Among these, the Co/NC catalyst demonstrated superior performance in OA hydrotreatment, achieving 99.6% conversion and 99.6% alkane selectivity, significantly outperforming Co/AC (76.8% conversion, 14.9% alkane) and Co/Al_2_O_3_ (96.3% conversion, 18.7% alkane). The NC support likely enhanced catalytic activity and stability, highlighting its potential for efficient biofuel production.

**Fig. 20:**
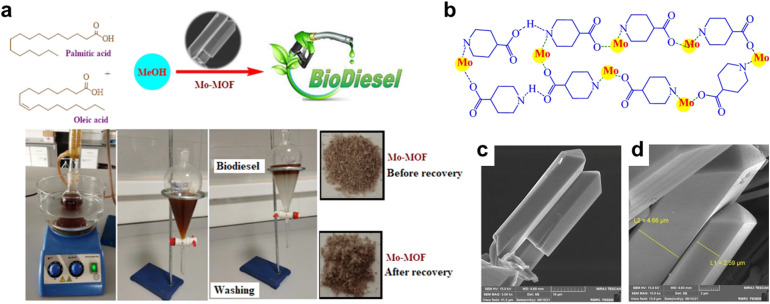
(a) Schematic of the application of Mo-MOF for producing biodiesel. (b) Topological structure of Mo–MOF. (c and d) SEM of Mo–MOF. This figure has been adapted/reproduced from ref. 252 with permission from Springer Nature, Copyright 2022.

While molybdenum-MOF (Mo-MOF) catalysts show great promise, it is important to consider the broader context of MOF-based catalysts in biodiesel production. The use of Mo-MOFs aligns with the goals of sustainable and eco-friendly biodiesel production. Their reusability and efficiency contribute to reducing waste and the need for additional resources.^76^ A. G. Choghamarani *et al.* synthesized a rod-like Mo-MOF catalyst *via* a solvothermal approach using piperidine-4-carboxylic acid, applying it to esterify oleic and palmitic acids for biodiesel fabrication.^252^ The catalyst achieved a high 95% biodiesel yield and exhibited robust reusability, retaining efficiency over four recovery cycles alongside thermal stability up to 300 °C. Fig. 20a schematically depicts the esterification process, illustrating the catalyst's role in converting fatty acids to biodiesel. Fig. 20b details the MOF's rod-like topology, emphasizing how metal ions and organic linkers form a structured network influenced by synthesis parameters (*e.g.*, solvent, temperature). Fig. 20c and d showcases SEM images of the catalyst, revealing uniform rod-shaped morphologies (500 nm–2 µm) with transversal alignment and monoclinic cleavage patterns, confirming its crystalline integrity and structural stability.

The use of Hf-MOF (Hafnium-based MOF) catalysts in biodiesel fabrication is an emerging area of research that leverages the unique properties of MOFs to enhance catalytic efficiency.^253^ R. Buzo *et al.* developed Hf-MOF for hydrogenation by catalytic transfer of levulinic ester to γ-valerolactone, achieving a 75% conversion.^254^ F. M. Mirante *et al.* demonstrated that MOF-808(Hf), serves as a highly efficient heterogeneous catalyst for the valorization of Gly, a byproduct of biodiesel production, *via* acetalization to synthesize solketal.^255^ Under optimized conditions (4 wt% catalyst loading, 1 : 6 Gly/acetone molar ratio, 333 K), the catalyst exhibited exceptional performance, achieving >90% Gly conversion and 98% solketal selectivity. The catalytic activity stems from MOF-808(Hf)'s high density of accessible acid sites and robust structural framework, which enables sustained activity over ten consecutive reaction cycles without significant degradation. This reusability, coupled with its thermal and chemical stability, underscores the catalyst's sustainability and potential for industrial applications in Gly upgrading.

B. B. Kulkarni *et al.* introduced a bifunctional Pd@UiO-66(Hf) core–shell MOF catalyst for the one-pot hydrogenation-esterification (OHE) of furfural with acetic acid, achieving selective production of furfuryl acetate.^256^ Compared to Pd/UiO-66(Hf) and Pd@UiO-66(Zr), the Pd@UiO-66(Hf) hybrid exhibited superior catalytic performance, with 82.7% furfural conversion and 68% selectivity toward furfuryl acetate. This efficiency arises from synergistic interactions between palladium nanoparticles (Pd NPs) and acidic sites on the UiO-66(Hf) secondary building units (SBUs), which facilitate tandem hydrogenation and esterification steps. Notably, this work represents the first application of MOF-based catalysts for OHE reactions in furfural upgrading, offering a sustainable strategy for converting biomass-derived molecules into high-value chemicals. The catalyst's design highlights the potential of multifunctional MOFs in cascade reactions for bio-oil valorization.

The incorporation of Ni into MOFs enhances their catalytic activity, making them suitable for both ester/transesterification processes essential for biodiesel synthesis. In a related investigation, Q. Zhang *et al.* synthesised HPMo/Ni-MOF for the production of biodiesel from fatty acid esterification, achieving an 86.10% conversion rate and good recyclability (activity at 73.5% after 10 cycles).^257^ In order to generate biodiesel and hydrogen from waste oils, W. Cong *et al.* created magnetic Ni-MOF *via* self-assembly.^258^ After five cycles, they achieved excellent recyclability and a 95.3% biodiesel conversion in 15 minutes when heated in a microwave at 90 °C.

A Ni-carbon composite catalyst (Ni@C500), derived from pyrolyzed Ni-based MOFs (Ni-BTC), was developed to enhance the deoxygenation and cracking of microalgal biodiesel for jet fuel-range hydrocarbon production.^259^ The pyrolysis process transformed coordinated Ni ions into highly active Ni nanoparticles while increasing the catalyst's specific surface area to improve mass transfer. X-ray absorption fine structure (XAFS) analysis confirmed the formation of Ni–Ni active sites, and DFT calculations identified C=C bonds as the primary cracking sites for long-chain fatty acids. At a catalyst-to-reactant mass ratio of 1 : 200, Ni@C500 achieved 97.46% conversion efficiency and 71.46% selectivity toward jet fuel-range hydrocarbons in methyl palmitate conversion, demonstrating its superior performance in microalgal biodiesel upgrading with minimal catalyst loading. This approach highlights the potential of MOF-derived catalysts for efficient, sustainable bio-jet fuel production.

Zr-MOFs, including derivatives and composites, are sustainable, high-performance catalysts for biodiesel production, excelling in trans/esterification due to their robust structure, reusability, and minimized environmental impact. Zr-MOFs, such as MOF-801, have demonstrated moderate catalytic activity in the transesterification of used vegetable oil, achieving a conversion rate of approximately 60% under specific conditions (50 wt% methanol: oil, 10 wt% catalyst loading, 180 °C, and 8 hours).^260^ Zr-MOFs exhibit good reusability, maintaining catalytic activity over multiple cycles. For instance, MOF-801 showed only a slight reduction in yield (∼10%) after three cycles, indicating its potential for repeated use without significant loss of activity.

In other studies, Q. Zhang *et al.* synthesized ZrSiW/Fe-BTC and ZrSiW/UiO-66 catalysts for biodiesel production, achieving 98% conversion with ZrSiW/UiO-66 outperforming ZrSiW/Fe-BTC (85.5%).^73^ Finally, the HPW@CoCeO catalyst was synthesized for methyl oleate production, achieving a 67.2% oil conversion after 4 hours at 60 °C, with good reusability.^261^ In another study, they synthesized HSiW@Ni–Zr–O-1 for biodiesel production, achieving a 95.2% oil conversion at 140 °C in 3 hours and maintaining 79.1% catalytic activity after 9 cycles.^262^ These studies demonstrate the high catalytic activity, recyclability, and potential for large-scale applications of MOF-based catalysts, underscoring their adaptability and efficiency in the production of biodiesel.

H. M. Salem *et al.* explored the hydrodeoxygenation (HDO) of OA, a renewable feedstock derived from non-edible and waste oils, to produce diesel-like hydrocarbons.^263^ A novel Zr-MOF/SBA-3 hybrid catalyst was designed to synergize the active sites of Zr-MOF with the ordered porosity of SBA-3, enhancing thermal stability and acid site distribution. Under optimized conditions (360 °C, atmospheric pressure, 10 h), the catalyst achieved 91.6% hydrocarbon selectivity, with kinetic analysis revealing an activation energy of 120 kJ mol^−1^ for OA conversion. The results demonstrate the potential of Zr-MOF/SBA-3 as an efficient catalyst for sustainable biofuel production, offering a scalable pathway to convert biomass-derived fatty acids into high-value hydrocarbon fuels.

## 7 Optimization and synergism of MOF catalysts for biodiesel production

To achieve good performance in biodiesel production using MOFs or MOF composites, several key material features must be considered. These features are critical for optimizing catalytic activity, stability, selectivity, and overall efficiency in the transesterification or esterification reactions required for biodiesel synthesis. Upon the current study, below is a detailed breakdown of the necessary MOF material characteristics and design considerations.

### 7.1 Engineered high-activity MOF catalysts

Engineered high-activity MOF catalysts for biodiesel production are designed through strategic incorporation of accessible and well-dispersed catalytic active sites, such as Lewis or Brønsted acid/base centers, which promote transesterification/esterification reactions between TGAs and alcohols.^120,156,264^ Catalytic activity is introduced *via* metal nodes (*e.g.*, Zn, Fe, Co, Al, Sn) or functionalized organic linkers, with redox-active metals (*e.g.*, Fe, Cu) selected for oxidative esterification and mixed-metal nodes employed to fine-tune acidity/basicity. An acid/base site density of 1–2 mmol g^−1^ total acidity is typically required for efficient FFA esterification, while 0.5–1.0 mmol g^−1^ basic sites ensures effective TAG transesterification, as confirmed through NH_3_-TPD and CO_2_-TPD analyses. Catalysts must also exhibit hierarchical porosity with mesopores ≥ 10–20 nm, which facilitates triglyceride diffusion and minimizes internal mass-transfer limitations. For feeds containing both FFAs and neutral oils, the acid-to-base site ratio should range between 3 : 1 and 1 : 1 to maximize bifunctional synergy in high-FFA systems, while a more basic ratio (<1 : 2) is preferable for neutral triglyceride oils. Additionally, maintaining a hydrophobic surface (water contact angle > 90°) enhances tolerance to wet or impure feedstocks by mitigating water adsorption and deactivation. Functionalization of MOFs further enhances catalytic performance through the introduction of acidic/basic functional groups (*e.g.*, –SO_3_H, –NH_2_, –COOH) or metal active centers (*e.g.*, Zr, Co), either *via* pre-synthetic linker modification or post-synthetic incorporation of catalytic species or immobilization of enzymatic species (*e.g.*, lipases), further amplifying selectivity.^31,118^ For instance, sulfonated MOFs (*e.g.*, MIL-101-SO_3_H) provide strong Brønsted acid sites, while composite strategies, such as combining MOFs with conductive supports (*e.g.*, graphene), improve reactant transport and mass transfer efficiency. These design principles collectively maximize catalytic activity, selectivity, and stability in biodiesel synthesis.^118,120^ Representative examples satisfying these criteria include UiO-66-SO_3_H (acidic, ∼1.25 mmol g^−1^) and CaO@ZnO composites derived from Ca-MOF precursors (basic, ∼0.8 mmol g^−1^), which have achieved > 95% conversion of OA and soybean oil, respectively.^11,265^

### 7.2 Robust MOF stability

Achieving robust structural stability is paramount for MOF catalysts in biodiesel production, enabling sustained performance under harsh conditions including high temperatures (>300 °C, with >80% TGA residual mass), humidity, acidic/alkaline environments, and polar solvents like methanol and water. Optimal frameworks, such as UiO-66(Zr) and MIL-101(Fe), exhibit hydrolytic stability constants >10^4^ M^−1^ (verified *via* DFT-predicted metal–ligand binding energies or hydrolysis tests), strong coordination for thermal endurance, and reusability over ≥10 cycles with <10% activity loss, preserving XRD crystallinity and surface area.^133,156^ Enhancements *via* hydrophobic linker modifications (*e.g.*, –CH_3_ or –F substitution) mitigate water-induced degradation, while composites with carbon-based supports (*e.g.*, graphene) or metal oxides bolster mechanical rigidity and recyclability, providing a roadmap for industrial viability.^108^

### 7.3 Precision-tuned MOF selectivity

Precision-tuned selectivity in MOF catalysts for biodiesel production is achieved *via* rational design of pore geometry (>2 nm apertures for triglyceride diffusion, <40 nm to exclude glycerol), acid/base site balance (separation >0.5 nm *via* DFT/EXAFS to suppress saponification <5%), and hydrophobic host–guest interactions that favor non-polar substrate adsorption over polar byproducts, enhancing ester yields by 20–30%.^122,265^ Frameworks like UiO-66(Zr) and ZIF-8 exemplify these principles, enabling site isolation, phase separation, and minimal side reactions for high-purity output.^266^

### 7.4 Reusability and recyclability

Ensuring catalyst longevity in MOF-based biodiesel production requires >90% activity retention over ≥5 cycles with <1 wt% metal leaching (ICP-OES), facilitated by defect-tolerant designs, cross-linked ligands, and magnetic composites like Fe_3_O_4_@UiO-66-NH_2_ or TiO_2_@Fe_3_O_4_@ZIF-8 for <2-min recovery without structural changes (XRD/XPS).^267,268^ Anchoring to supports (*e.g.*, silica, carbon) enhances mechanical stability, minimizing waste and aligning with green chemistry for sustainable, circular processes.^108^

### 7.5 Sustainable, scalable, eco-friendly MOF solutions

For industrial deployment, MOF catalyst synthesis for biodiesel production must prioritize energy efficiency (<2 MJ kg^−1^*via* microwave-assisted or aqueous solvothermal routes), high solvent recovery (>80%), and cost-effectiveness (<10 USD kg^−1^ using abundant, non-toxic metals like Fe, Al, Zn, and recyclable linkers from plastic/biomass waste), reducing energy demand by 30–50% and solvent waste by >70% compared to conventional catalysts like sulfated zirconia or CaO.^66^ These eco-conscious strategies, exemplified by Fe-based MIL-100(Fe) frameworks, align with green chemistry principles, enabling scalable, low-impact production while minimizing environmental footprints.^156^

## 8 Future prospects for MOF catalysts in biodiesel production

Despite these advancements, challenges persist in MOF-based catalysts for biodiesel production, including stability issues under harsh conditions (*e.g.*, structural degradation, leaching, and sensitivity to high FFA/water content), limited reusability due to catalyst deactivation over cycles, scalability limitations from lab-scale synthesis to industrial batches, high synthesis costs and resource intensity (*e.g.*, reliance on rare metals or energy-demanding methods), gaps in mechanistic understanding and optimization for consistent high yields, and insufficient environmental/economic viability assessments like comprehensive life-cycle analyses.

Despite these advancements, challenges persist in MOF-based catalysts for biodiesel production, including stability issues under harsh conditions (*e.g.*, structural degradation, leaching, and sensitivity to high FFA/water content), limited reusability due to catalyst deactivation over cycles, scalability limitations from lab-scale synthesis to industrial batches, high synthesis costs and resource intensity (*e.g.*, reliance on rare metals or energy-demanding methods), gaps in mechanistic understanding and optimization for consistent high yields, and insufficient environmental/economic viability assessments like comprehensive life-cycle analyses. Life cycle assessments of MOF-catalyzed biodiesel production highlight reduced environmental impacts, with global warming potential as low as 0.21 kg CO_2_ eq kg^−1^ biodiesel due to catalyst reusability (>5 cycles), minimizing waste and energy use *versus* homogeneous systems (20–30% lower emissions overall).^269,270^

Looking ahead, these challenges underscore promising prospects for future research. Prioritizing underexplored MOF systems, such as Ca- and Cu-based frameworks, offer cost-effective and eco-friendly alternatives, while innovations in green synthesis methods utilizing abundant metals, recycled linkers from waste (*e.g.*, PET-derived), and energy-efficient techniques like microwave-assisted or solvent-free approaches can enhance scalability, sustainability, and reduce costs. Advanced functionalization strategies, including machine learning-guided design for tailored active sites (*e.g.*, dual Lewis–Brønsted acids), hierarchical structures, metal doping (*e.g.*, Fe(iii), Ce, Cr), and enzyme-MOF hybrids, could boost performance in handling diverse feedstocks like waste oils, with deeper mechanistic studies (*e.g.*, *via* XPS, kinetic modeling) enabling optimized reaction pathways under mild conditions. Addressing stability and reusability through robust composites, protective coatings, self-healing designs, or magnetically separable MOFs (*e.g.*, Fe@C–Sr, magnetized ZIF-8) will facilitate extended catalyst life and easier recycling. Integrating MOFs with emerging technologies, such as continuous-flow reactors and photothermal/microwave-assisted processes, holds potential for industrial-scale implementation.

In these lights, below some points to consider for future research: (1) Enhanced Structural Stability: Develop MOFs capable of retaining ≥90% crystallinity and >95% catalytic activity after exposure to methanol/water mixtures for 50 h, verified *via* XRD and BET analyses. (2) High Site Density Catalysts: Design frameworks with acid/base site densities of 1–2 mmol g^−1^, optimized through defect engineering or functionalization (–SO_3_H, –NH_2_), to achieve ≥98% FAME yield from high-FFA oils. (3) Hierarchical and Bifunctional Architectures:Construct MOFs with mesopores of 10–30 nm and acid: base ratios between 2:1–1:1 for one-pot conversion of mixed FFA/TAG feedstocks under mild conditions (<70 °C). (4) Green and Scalable Synthesis:Establish low-energy (<2 MJ kg^−1^) and solvent-efficient (>85% recovery) synthesis routes, such as microwave-assisted or solvent-free processes, validated through life-cycle assessment (LCA). (5) Recyclability and Zero-Leaching Systems: Achieve >90% activity retention over 10 cycles with <0.5 wt% metal leaching, confirmed by ICP-OES, using robust MOF-oxide or MOF-carbon composites. (6) Waste-Derived and Bio-Based Precursors: Utilize recycled linkers from PET or biomass waste and abundant metals (Fe, Al, Ca, Zn) to produce catalysts costing <10 USD kg^−1^, supporting circular-economy synthesis. (7) Mechanistic and *Operando* Insights: Combine techniques like XPS, EXAFS, and Py-IR with DFT modeling to quantify active-site evolution and measure reaction barriers within ± 10 kJ mol^−1^ of experimental data. (8) Integration into Continuous-Flow Systems: Validate MOF catalysts in continuous-flow reactors with throughput ≥1 L h^−1^ and steady conversion for ≥100 h to demonstrate process scalability. (9) Hybrid and Smart Catalytic Designs: Develop magnetically separable, self-healing, or photothermal-responsive MOF composites achieving catalyst recovery <2 min and ≥20% energy savings compared with conventional heating. (10) Comprehensive Techno-Economic and Environmental Evaluation:Conduct techno-economic analysis (TEA) and full life-cycle assessment (LCA) to confirm an energy return on investment (EROI) >3 and GHG reduction > 60% relative to petro-diesel benchmarks. (11) The combination between to unique features (hydrophobicity and multimetal sites) *via* one pot and green approach to apply it in various organic reaction especially, esterification or transesterification of free fatty acid and vegetable oils and other reactions that were occurring in aqueous medium.

## 9 Conclusion

In summary, this review underscores the transformative potential of MOFs as heterogeneous catalysts in advancing sustainable biodiesel production. MOFs with their exceptional attributes have emerged as superior alternatives to traditional homogeneous and heterogeneous catalysts. Through detailed examination of key MOF families, including UiO-series (*e.g.*, UiO-66 and its derivatives), MIL-series (*e.g.*, MIL-100 and MIL-101), ZIF-series (*e.g.*, ZIF-8 and ZIF-67), and others like Ca- and Cu-based MOFs, the review highlights significant progress in esterification and transesterification reactions. Functionalized MOFs, composites (*e.g.*, with metal oxides, polyoxometalates, or magnetic supports), bifunctional systems, and enzyme-MOF hybrids have demonstrated remarkable catalytic efficiencies, often achieving biodiesel yields exceeding 95% under optimized conditions, with enhanced reusability (up to 10 cycles) and stability against leaching, deactivation, and harsh environments. Mechanistic insights reveal the synergistic roles of Lewis and Brønsted acid/base sites, while kinetic and thermodynamic studies confirm lower activation energies and favorable reaction pathways compared to conventional catalysts. Characterization techniques play a pivotal role in elucidating the underlying reaction mechanisms and assessing the long-term stability of catalysts. Moreover, the integration of MOFs aligns with circular economy principles by enabling the valorization of waste feedstocks like used cooking oils and acidic oils, reducing environmental impacts, and improving techno-economic viability through simplified separation and recycling.

## Author contributions

Basem E. Keshta: conceptualization, methodology, formal analysis, writing—original draft, visualization, supervision, writing—review and editing. Sahar Abdalla: funding acquisition, validation, investigation. Dhiss Tesnim: writing—review and editing, validation; Yasmeen G. Abou El-Reash: investigation, resources, data curation; Eida S. Al-Farraj: formal analysis, investigation; Mahmoud M. Shaban: writing—original draft, visualization, formal analysis. Ahmed El-Harairy: writing—original draft, visualization, formal analysis. H.M. El-Saeed: writing—original draft, visualization, formal analysis. Amged El-Harairy: writing—original draft, visualization, formal analysis. Ebtehal A. Shaban: writing—original draft, visualization, formal analysis. Ahmed S. Abou-Elyazed: investigation, writing—original draft, writing—review and editing. Mohamed N. Goda: funding acquisition, validation, investigation. Elsayed M. Atwa: visualization, writing—review and editing. Amr E. Keshta: visualization, writing—review and editing.

## Conflicts of interest

There are no conflicts to declare.

## Data Availability

No data was used as part of this review.
